# Melatonin-Induced Modulation of Polyphenols and Glycolytic Pathways in Relation to Postharvest Quality of Blue Honeysuckle Fruits

**DOI:** 10.3390/foods14152646

**Published:** 2025-07-28

**Authors:** Jinli Qiao, Liangchuan Guo, Zhen Xiao, Junwei Huo, Xiaonan Sui, Fang Gao, Yan Zhang

**Affiliations:** 1 Heilongjiang Green Food Science Research Institute, Northeast Agricultural University, Harbin 150030, China; 2 College of Horticulture and Landscape Architecture, Northeast Agricultural University, Harbin 150030, China; b220401011@neau.edu.cn (J.Q.);; 3 National-Local Joint Engineering Research Center for Development and Utilization of Small Fruits in Cold Regions, Northeast Agricultural University, Harbin 150030, China; 4 College of Food Science, Northeast Agricultural University, Harbin 150030, China

**Keywords:** melatonin, blue honeysuckle, polyphenol, sugar, antioxidant capacity

## Abstract

The impact of exogenous melatonin treatment on the postharvest quality and storability of blue honeysuckle fruit was investigated. Fruits were immersed in melatonin solutions at concentrations of 0 (control), 0.01, 0.05, and 0.25 mM for 5 min and subsequently stored at –1 °C for 63 d. Among all treatments, the combination of two-week storage without fruit puncturing and 0.05 mM melatonin application significantly delayed fruit softening and decay even at the initial stage of storage, while also increasing the concentration of phenolic compounds and enhancing antioxidant activity. During the later storage period (28–63 d), melatonin-treated fruits maintained higher levels of maltose, fructose, and sucrose, contributing to improved flavor retention. In contrast, both lower (0.01 mM) and higher (0.25 mM) concentrations were less effective or even detrimental to fruit quality. HPLC-ESI-QTOF-MS^2^ analysis revealed that 0.05 mM melatonin effectively preserved several functional phenolics, including p-coumaroylquinic acid, caffeoyl glucose, 5-O-caffeoylquinic acid, 3-O-caffeoylquinic acid, luteolin-7-O-glucoside, and hydroxytyrosol. Thus, 0.05 mM melatonin is effective in delaying senescence and maintaining the postharvest quality of blue honeysuckle fruit.

## 1. Introduction

Blue honeysuckle (*Lonicera caerulea* L.), a widely consumed berry crop, is well regarded by consumers for its pleasant taste and high nutritional value, particularly its abundance of organic acids, anthocyanins, and polyphenols [1]. The major polyphenols in blue honeysuckle include anthocyanins (particularly cyanidin-3-glucoside and cyanidin-3,5-diglucoside), flavonols and isoflavonols (quercetin-3-rutinoside), flavanols (epicatechin), flavones (apigenin-7-rutinoside), and phenolic acids (chlorogenic acid) [2]. Among these, anthocyanins are the most abundant and are responsible for the deep purple color of the fruit. These compounds have been reported to exhibit various biological activities, including anti-inflammatory, anti-diabetic, antioxidant, and cardioprotective effects [1]. While previous studies have primarily focused on comparing the polyphenol content among different blue honeysuckle cultivars, little is known about postharvest changes in these compounds, leaving a significant research gap in this area. After harvest, the fruits are disconnected from the parent plant’s water and nutrient supply, triggering endogenous metabolic changes and self-degradation processes [3]. As a non-climacteric berry, blue honeysuckle is prone to rapid softening, nutrient loss, and fluctuations in sugar content during storage—issues that are difficult to avoid and result in diminished nutritional quality, poor flavor, reduced consumer acceptance, and economic losses [4]. Various postharvest strategies, such as low-temperature storage [4], aloe vera–chitosan composite coatings [5], and controlled atmosphere techniques [6], have been employed to preserve fruit quality. However, some of these methods either lack a focus on shelf-life extension or are not commercially viable due to limited consumer acceptance or insufficient validation. Therefore, enhancing quality retention and extending the storage life of blue honeysuckle remain pressing challenges for the industry.

Melatonin is a regulatory molecule that is widely distributed across various fruit species [7]. It has been reported to delay quality deterioration by preserving firmness, acidity, and soluble solids, while also reducing the respiration rate during fruit ripening [3]. Postharvest applications of melatonin in strawberries, grapes, kiwifruits, and other fruits have shown effectiveness in delaying softening by modulating key metabolic pathways, including polyphenolic metabolism, glycolytic, cell wall degradation, ascorbic acid metabolism, and reactive oxygen species (ROS) scavenging pathways [8,9]. Melatonin treatment upregulates the activity of enzymes involved in phenylpropanoid metabolism, such as PAL, CHS, and DFR, thereby inducing the biosynthesis of anthocyanins and other flavonoids. Additionally, melatonin influences carbohydrate metabolism and energy homeostasis by regulating glycolysis-related enzymes, thus maintaining membrane integrity and fruit quality during storage. In melatonin-treated plants, elevated levels of proline and carbohydrates, including glucose, maltose, fructose, sucrose, and trehalose, have been reported under abiotic stress conditions, further contributing to osmotic regulation, energy supply, and enhanced stress tolerance during postharvest storage [9]. These effects contribute to extended storage life and improved product quality, highlighting melatonin’s promising potential for commercial use [10,11].

This study aimed to evaluate the effects of exogenous melatonin on the postharvest performance of blue honeysuckle, with a focus on changes in firmness, sugar and organic acid content, polyphenol accumulation, and antioxidant capacity during storage. The findings provide new insights into the regulatory role of melatonin in preserving the postharvest quality of blue honeysuckle fruit.

## 2. Materials and Methods

### 2.1. Chemicals

Standard compounds—including sucrose, maltose, glucose, fructose, malic acid, citric acid, gallic acid, ferrous sulfate heptahydrate, Trolox—were purchased from Yuanye (Shanghai, China). Melatonin was obtained from Solarbio (Beijing, China). HPLC-grade potassium dihydrogen phosphate, methanol, and acetonitrile were purchased from Xingmake (Tianjin, China), and MS-grade acetonitrile, formic acid, and ammonium acetate came from Merck (Darmstadt, Germany). Chemical reagents such as hydrochloric acid, Folin–Ciocalteu reagent, sodium carbonate, glacial acetic acid, ABTS (2,2′-azino-bis(3-ethylbenzothiazoline-6-sulfonic acid) diamine salt), sodium hydroxide, potassium chloride, ferric chloride hexahydrate, DPPH (2,2-diphenyl-1-picrylhydrazyl), and sodium acetate were purchased from Kemio (Tianjin, China).

### 2.2. Sample Treatment

Fresh blue honeysuckle “Lanjingling” was selected based on maturity and soluble solid content. The fruit was sourced from the Xiangyang experimental farm of the Northeast Agricultural University Xiangyang Experimental Farm in Harbin, China. For the experiment, 20 kg of intact blue honeysuckle fruit free from mechanical injury, pests, and diseases was carefully selected. The fruits were evenly randomized into four treatment groups, each comprising three biological replicates of 5 kg. The treatments included a control (sterile water) and melatonin solutions at concentrations of 0.01 mM, 0.05 mM, and 0.25 mM. Blue honeysuckle samples were submerged in the respective solutions for 5 min, then transferred into sterilized polyethylene terephthalate (PET) containers, with each treatment replicated three times. Following immersion, the samples were air-dried at ambient temperature and subsequently stored at –1 °C under 90% relative humidity. Initial assessments were conducted on the day storage began, followed by sample collections on days 3, 7, 14, 21, 28, 35, 49, and 63 to evaluate various quality parameters.

### 2.3. Surface Color

A CR-400 chroma meter (Konica Minolta Inc., Osaka, Japan) with a 6 mm aperture, 0° viewing angle, and illuminant C was used to evaluate the color attributes of blue honeysuckle. A white calibration cap was used to standardize the colorimeter, and color was described using the L*, a*, b* color system defined by the International Commission on Illumination (CIE) [12].

The chroma was determined using the following formula:

(1) $$Chroma\left(C\right)=\sqrt{\left(a^{{*}^{2}}+b^{{*}^{2}}\right)}$$

The hue angle was calculated based on

(2) $$Hue\;angle\left(h\right)=arctan\frac{b^{*}}{a^{*}}$$

To evaluate changes in fruit surface color across different treatments and storage times, the total color difference (ΔE) was computed using

(3) ∆E=L*sample−L*02+a*0−a*02+b*sample−b*0212

where ΔE indicates chromatic aberration; L*sample, a*sample, and b*sample are post-storage values, while L*_0_, a*_0_, and b*_0_ represent measurements at harvest.

### 2.4. Weight Loss and Firmness

Weight loss was calculated using the following formula:

(4) $$Weight\;loss\left(\%\right)=\frac{W_{0}-W}{W_{0}}\times 100\%$$

where *W*_0_ and *Wₜ* correspond to the initial weight and the weight at day 63, respectively.

Using a hardness tester (FHM-5, Takemura Electric Work Ltd., Japan) with an 8 mm probe, firmness was measured by gently lowering the probe to the bottom of the blue honeysuckle and recording the value at that point.

### 2.5. Sugars and Total Soluble Solids

Sugars, including sucrose, maltose, glucose, and fructose, were separated using a Hi-Plex Ca column (300 × 7.7 mm, Agilent, Santa Clara, CA, USA) on an HPLC system (1260 Infinity II, Agilent Technologies, Santa Clara, CA, USA). Ultrapure water served as the mobile phase and was monitored using a refractive index detector (1260 RID, Agilent) operated at 80 °C, with a flow rate set to 0.4 mL/min [13]. Total soluble solids (TSS) were determined with a PAL-BX/ACID7 handheld Brix-acidity meter (ATAGO, Tokyo, Japan).

### 2.6. Organic Acids, Acidity, and pH

Separation of malic and citric acids was performed on a Zorbax SB-Aq column (4.6 × 150 mm, Agilent, USA) on an HPLC system (1260 Infinity II, Agilent Technologies, CA, USA). A phosphate buffer (pH 2.0) containing 1% acetonitrile was employed as the eluent. Detection was carried out with a diode array detector (DAD, 1260 DAD, Agilent) at a wavelength of 210 nm [14]. Acidity and pH levels were measured using a Brix-acidity meter (PAL-BXIACID F5, ATAGO, Japan) and a pH meter (Fiveeasy plus FE28, Mettler Toledo, Shanghai, China), respectively.

### 2.7. Determination of Total Phenolic Content (TPC), Total Anthocyanin Content (TAC), and Total Flavonoid Content (TFC)

The fruit samples were first ground into fine powder using liquid nitrogen. Then, 2.0 g of homogenized sample was mixed with 10 mL of 80% methanol (*v*/*v*). The mixture was placed on a shaker (SPH-2102C, Shiping, Shanghai, China) at 25 °C for 24 h to ensure sufficient extraction. After incubation, the extract was centrifuged at 8000 rpm for 10 min at 4 °C, and the supernatant was collected for TPC, TAC, and TFC analysis.

TPC was quantified employing the method described by Singleton et al. [15]. A gradient-diluted sample was initially aliquoted (20 μL) into a 96-well microplate, followed by the addition of 90 μL of water and 10 μL of Folin–Ciocalteu reagent. The reaction mixture was subsequently incubated in the absence of light at 25 °C for 5 min. Thereafter, 80 μL of sodium carbonate solution (75 g·L^−1^) was introduced, and the plate was gently agitated to ensure homogeneous mixing. Absorbance readings were subsequently taken at 765 nm using a microplate spectrophotometer (Epoch 2, BioTek, Winooski, VT, USA).

TAC was determined according to Giusti and Wrolstad [16]. Briefly, aliquots were diluted separately in potassium chloride (0.025 M) and sodium acetate buffers (0.4 M), respectively. Absorbance was then measured at 510 nm and 700 nm using a microplate spectrophotometer (Epoch 2, BioTek, USA).

TFC was evaluated through a spectrophotometric colorimetric assay utilizing aluminum chloride, based on the methodology outlined by Chang et al. [17]. Specifically, a 30 μL test solution was pipetted into a 96-well microplate and combined with 180 μL of deionized water and 10 μL of 5% (*v*/*v*) sodium nitrite. The resulting reaction system was incubated at 25 °C for 6 min. Then, 20 μL of 10% (*v*/*v*) AlCl_3_ was administered, and the contents were gently vortexed. After another 6 min reaction period, 60 μL of 4% sodium hydroxide was added, and the mixture was subjected to incubation at 37 °C for 15 min. Absorbance was subsequently monitored at 510 nm using a microplate spectrophotometer (Epoch 2, BioTek, USA).

### 2.8. Characterization and Quantification of Polyphenols

The polyphenol extract used for HPLC-ESI-QTOF-MS^2^ analysis was extracted using the same procedure as described in Section 2.7. Polyphenol profiling was conducted via HPLC-PDA and HPLC-ESI-QTOF-MS^2^ (AB Sciex, Redwood City, CA, USA), employing a C18 reverse-phase analytical column (Luna, 5 μm, 250 × 4.6 mm; Phenomenex, Torrance, CA, USA). The elution system consisted of water containing 60 mM formic acid (solvent A) and acetonitrile with 5 mM aqueous ammonium acetate (solvent B). The chromatographic gradient elution program was as follows: 14–16.5% solvent B (0–12.5 min), 16.5–25% B (12.5–17.5 min), 25–80% B (17.5–40 min), 80–50% B (40–55 min), and 50–14% B (55–60 min), followed by a re-equilibration phase at 5% B for 5 min. The column compartment was maintained at 25 °C, the flow rate was 0.8 mL/min, and the injection volume was 1 µL. Data acquisition was performed at absorbance wavelengths of 280 nm and 520 nm, respectively, to enable targeted detection of polyphenols and anthocyanins [2]. The mass spectrometer was operated to acquire full-scan spectra over *m/z* 100–2000 (MS) and *m/z* 50–2000 (MS^2^) in both positive and negative ion modes. Negative ion electrospray ionization (ESI) was performed with a capillary voltage of 4500 V, whereas positive ion ESI employed a capillary voltage of 5500 V, with the desolvation gas temperature maintained at 550 °C. The curtain gas was set to 35 psi, and the drying gas temperature was also maintained at 550 °C. For MS^2^ fragmentation, the collision energy spread was set to 20 V.

### 2.9. Antioxidant Capacity

The DPPH was evaluated following the protocol described by Brand-Williams et al. [18]. Briefly, 5 μL of the appropriately diluted sample was combined with 195 μL of DPPH solution and vortexed thoroughly. The resulting mixture was recorded at 515 nm with a microplate spectrophotometer. Trolox was used to construct a calibration curve at concentrations of 100–1000 μmol/L. The standard curve was linear with R^2^ = 0.9943 (y=0.0522x−8.6889). The ABTS was evaluated according to the methodology established by Re et al. [19]. The sample (10 μL) was mixed with 190 μL of ABTS^+^ working reagent (0.70 ± 0.01 at 734 nm), and the absorbance was determined at 734 nm with a microplate spectrophotometer. Trolox solutions (50–800 μmol/L) were used to prepare the standard curve (y=0.0891x+6.3275, R^2^ = 0.9971). DPPH and ABTS were calculated with the following formula:

(5) $$y\left(\%\right)=1-\left(\frac{{OD}_{1}}{{OD}_{0}}\right)\times 100\%$$

where OD_1_ is the absorbance of the sample or Trolox, and OD_0_ is the absorbance of the control. The calculated Y values were then substituted into the Trolox standard calibration curve to determine the antioxidant capacity.

The FRAP was conducted following the procedure described by Benzie and Strain [20]. The FRAP reagent (solution B) was freshly formulated by blending 10 mM TPTZ, 20 mM FeCl_3_·6H_2_O, and 300 mM acetate buffer in a volumetric ratio of 1:1:10. An aliquot of 150 μL of solution B, 10 μL of the prepared sample, and 30 μL of distilled water were pipetted into a 96-well microplate. The absorbance was determined at 593 nm with a microplate spectrophotometer. The standard curve was generated using FeSO_4_·7H_2_O (100–1000 μmol/L), with R^2^ = 0.9918 (y=0.004x+0.0086).

### 2.10. MDA Content

The measurement of malondialdehyde (MDA) was conducted following the method outlined by Li et al. [21]. The absorbance was recorded at three wavelengths (450, 532, 600 nm) via a spectrophotometer (UV-1800, Shimadzu Corporation, Kyoto, Japan).

### 2.11. Statistical Analysis

Statistical analyses were conducted using one-way ANOVA, with significant differences determined at *p* ≤ 0.05 by Duncan’s multiple range test. All measurements were performed in triplicate. The data processing was performed using IBM SPSS Statistics version 26 (SPSS Inc., Chicago, IL, USA). For multivariate data exploration, principal component analysis (PCA) and Pearson correlation coefficients were calculated utilizing OriginLab Origin 2021 software (OriginLab, MicroCal, MA, USA). The PCA dataset was auto-scaled (mean-centered and divided by standard deviation) using a built-in preprocessing function to normalize the variables in Origin. Pearson correlation coefficients were calculated using Origin’s correlation matrix function. The resulting correlation matrix was visualized as a heatmap generated with the built-in correlation plot tool.

## 3. Results and Discussion

### 3.1. Appearance and Color of Blue Honeysuckle Fruits

**Figure 1:**
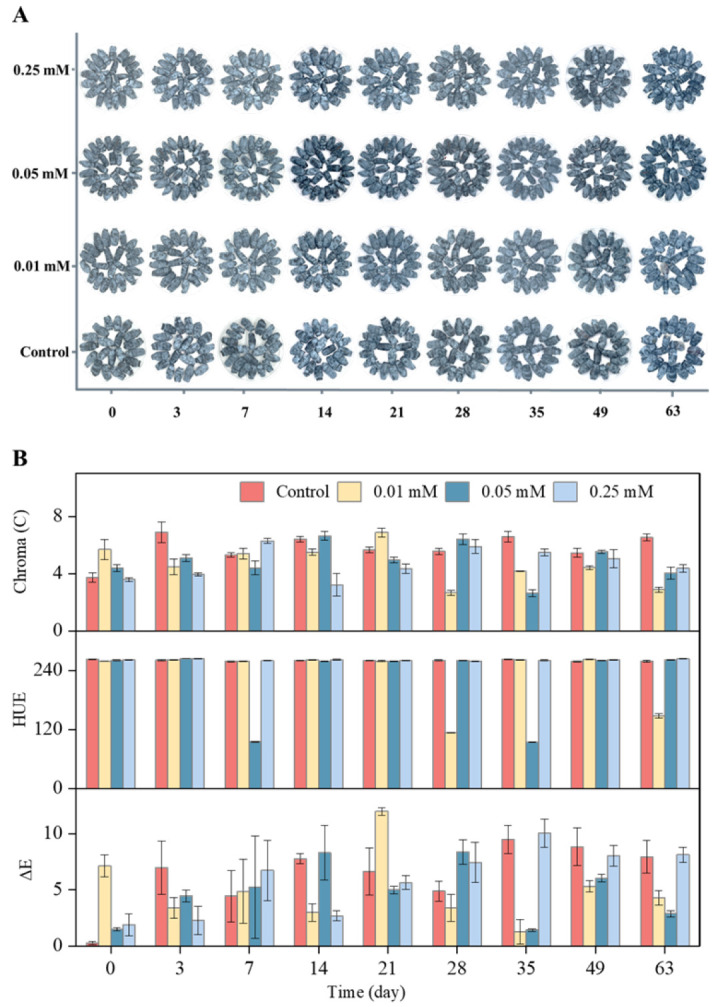
Visual fruit color (**A**), chroma, hue, and color difference (ΔE) (**B**) of control and melatonin (0.01, 0.05, 0.25 mM) in blue honeysuckle during storage for 63 d. Error bars represent the standard deviation of the mean of three replicates.

Color plays a crucial role in shaping consumer perception [5], and noticeable visual changes in blue honeysuckle were observed during storage (Figure 1A). By day 49, fruits in the control group began to exhibit slight decay, whereas those treated with melatonin showed visible signs of decay only after 63 d, indicating that melatonin effectively extended shelf life by approximately 14 d. Initial color values—including L*, a*, b*, chroma, hue angle, and color difference—were 26.39, −0.47, −3.7, 3.73, 262.68, and 0.25, respectively (Table A1, Figure 1B). Notably, lower chroma values in this context correspond to more vivid fruit coloration. Melatonin treatment significantly improved color stability during storage. After 63 d, fruits treated with low doses of melatonin exhibited reduced chroma values, resulting in more vibrant color compared to the control. These results suggest that low-dose melatonin helps to maintain visual quality and extends shelf life, consistent with findings reported by Wu et al. in yellow peaches [22]. While hue angle remained relatively stable across treatments, color difference varied, with 0.05 mM melatonin producing the smallest chromatic deviation by day 63. Overall, melatonin was effective in preserving the appearance and prolonging the postharvest longevity of blue honeysuckle.

### 3.2. Weight Loss and Fruit Firmness of Blue Honeysuckle Fruits

Weight loss and firmness are critical indicators of fruit quality during storage, as they directly affect flavor, texture, sensory attributes, marketability, and shelf life [23]. Weight loss reflects moisture loss and is closely associated with fruit freshness over time. As shown in Table A1, fruit weight loss progressively increased with storage duration, likely due to respiration-induced water loss [4]. Interestingly, melatonin treatment led to a greater weight loss compared to the control, with this effect becoming more pronounced at higher concentrations throughout the storage period. This increase may be attributed to melatonin’s influence on epidermal moisture regulation, potentially through changes in stomatal behavior or peel structure, thereby promoting moisture evaporation [23].

Firmness, a key determinant of fruit texture, initially declined after harvest and then fluctuated, dropping from 1.31 N (Table A1). On days 21, 28, and 63, fruits treated with melatonin exhibited significantly higher firmness than the control, indicating its role in preserving texture. Furthermore, melatonin delayed softening in a dose-dependent manner, with 0.05 mM and 0.25 mM treatments proving more effective than the lower dose. Similar trends have been reported in peaches, where higher melatonin concentrations help to regulate ripening and maintain firmness postharvest [22].

### 3.3. Sugars and Acids of Blue Honeysuckle Fruits

**Figure 2:**
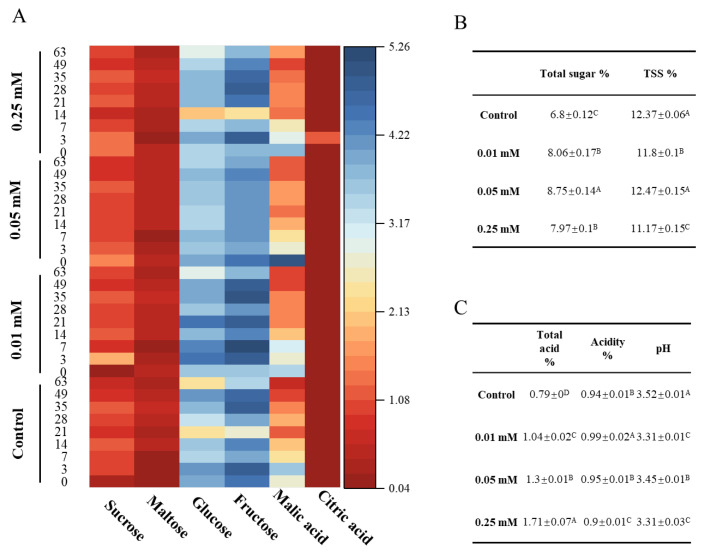
Sugar (sucrose, maltose, glucose, and fructose) and acid (malic acid and citric acid) of control and melatonin (0.01, 0.05, 0.25 mM) in blue honeysuckle during storage for 63 d (**A**). Total sugar, TSS content (**B**), total acid, acidity, and pH (**C**) of blue honeysuckle in 63 d. Data are shown as the mean ± S.E. Different uppercase letters in (**B**,**C**) indicate significant differences among treatments (*p* < 0.05).

The concentration and composition of total soluble solids (TSS) are key determinants of fruit quality, contributing to energy storage, signaling, and osmoregulation [24]. Treatment with 0.05 mM melatonin significantly increased soluble sugar levels compared to the control, whereas 0.25 mM had a relatively weaker effect (Figure 2A,B; Table A1). The primary sugars in blue honeysuckle include sucrose, maltose, glucose, and fructose, with glucose and fructose present in nearly equal amounts. By day 63, 0.05 mM melatonin elevated the contents of sucrose, maltose, glucose, and fructose by 1.28, 1.38, 1.44, and 1.16-fold, respectively, relative to the control. Although sugar levels initially increased and later declined during storage, treatments with 0.01 mM and 0.05 mM melatonin helped to stabilize fructose and glucose accumulation. These results are partially consistent with previous findings in blueberries, where melatonin application maintained sugar levels and delayed senescence by regulating carbohydrate metabolism and enhancing stress tolerance [25]. Increasing evidence suggests that elevated soluble sugar concentrations are crucial for preserving fruit quality, delaying senescence, and improving resistance to chilling stress during postharvest storage [24]. Melatonin has been shown to stimulate sucrose biosynthesis and its hydrolysis into glucose and fructose, underscoring its key role in carbohydrate metabolism [26].

Acidity is another critical factor in preserving the postharvest flavor of blue honeysuckle. In this study, organic acid content declined gradually over the storage period (Figure 2A,C; Table A1). However, by day 63, fruits treated with 0.01 mM melatonin retained significantly higher organic acid content than the control. Maintaining adequate levels of organic acids and soluble solids is essential for preserving the sugar–acid balance, which is fundamental to overall fruit quality during storage [24]. HPLC analysis identified malic and citric acids as the predominant organic acids in blue honeysuckle. Exogenous melatonin significantly increased malic acid levels, with higher concentrations inducing greater accumulation. This suggests that melatonin enhances malic acid biosynthesis, thereby reducing acid loss and contributing to fruit quality preservation. This observation is consistent with Dou et al., who reported increased malic acid levels in melatonin-treated tomatoes [26].

### 3.4. TPC, TAC, and TFC of Blue Honeysuckle Fruits

**Figure 3:**
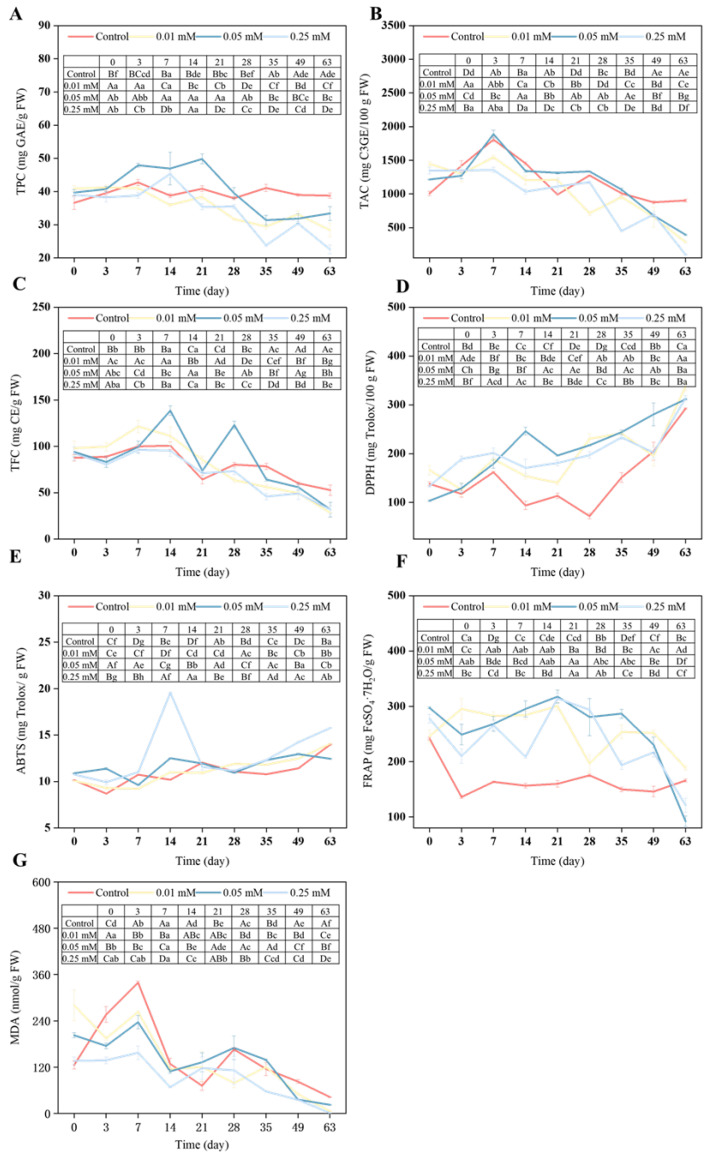
Changes in the (**A**) TPC, (**B**) TAC, (**C**) TFC, (**D**) DPPH, (**E**) ABTS, (**F**) FRAP, and (**G**) MDA of blue honeysuckle fruit stored at −1 °C. Values were expressed as gallic acid equivalent (GAE), catechin equivalent (CE), and cyanidin-3-glucoside equivalent (C3G). Different letters (lowercase: between different treatments; uppercase: between different storage days) indicate differences (*p* < 0.05).

Analysis of TPC, TAC, and TFC in postharvest blue honeysuckle revealed distinct patterns among treatment groups, with most variation occurring around day 35 of storage (Figure 3A). In the 0.05 mM melatonin treatment group, TPC levels were significantly higher than those of the control during the first 28 days. However, by day 63, the control group exhibited higher TPC levels than all treatment groups. This shift may be due to the gradual depletion of melatonin-induced antioxidant effects, combined with increased endogenous oxidative stress and polyphenol degradation during prolonged storage [25]. Additionally, enhanced phenolic synthesis in the control group may have been triggered by stress signaling in the later stages of storage. Regarding TAC, differences among treatments were observed throughout storage (Figure 3B). In the control group, TAC initially increased during early storage before gradually declining. In contrast, the 0.05 mM melatonin group consistently maintained higher TAC levels, particularly after day 14, with statistically significant increases compared to the control. By day 63, TAC had declined in all groups, and differences among treatments became negligible. TFC levels in both control and melatonin-treated fruits showed a transient increase followed by a gradual decline (Figure 3C). In early storage, TFC was significantly higher in melatonin-treated groups, with peak accumulation under 0.01 mM and 0.05 mM treatments. However, after day 28, TFC levels in the control group surpassed those in the treated groups. The early enhancement of antioxidant capacity by melatonin likely contributed to the stabilization of phenolics, anthocyanins, and flavonoids, resulting in higher initial levels. As storage progressed, the protective effects of melatonin diminished, while intrinsic metabolic activity, oxidative stress, and shifts in the internal environment accelerated degradation of these bioactive compounds, leading to lower levels in treated fruits compared to the control in the later stages. These findings are consistent with those of Li et al. [27], who reported that TPC in grapes initially increased within 4 d of melatonin treatment before falling below control levels by day 8. Overall, the data suggest that melatonin application, particularly at 0.05 mM, effectively delays polyphenol degradation during early storage, with the most pronounced benefits observed in the early postharvest period.

### 3.5. Qualitative Analysis of Polyphenols of Blue Honeysuckle Fruits

**Figure 4:**
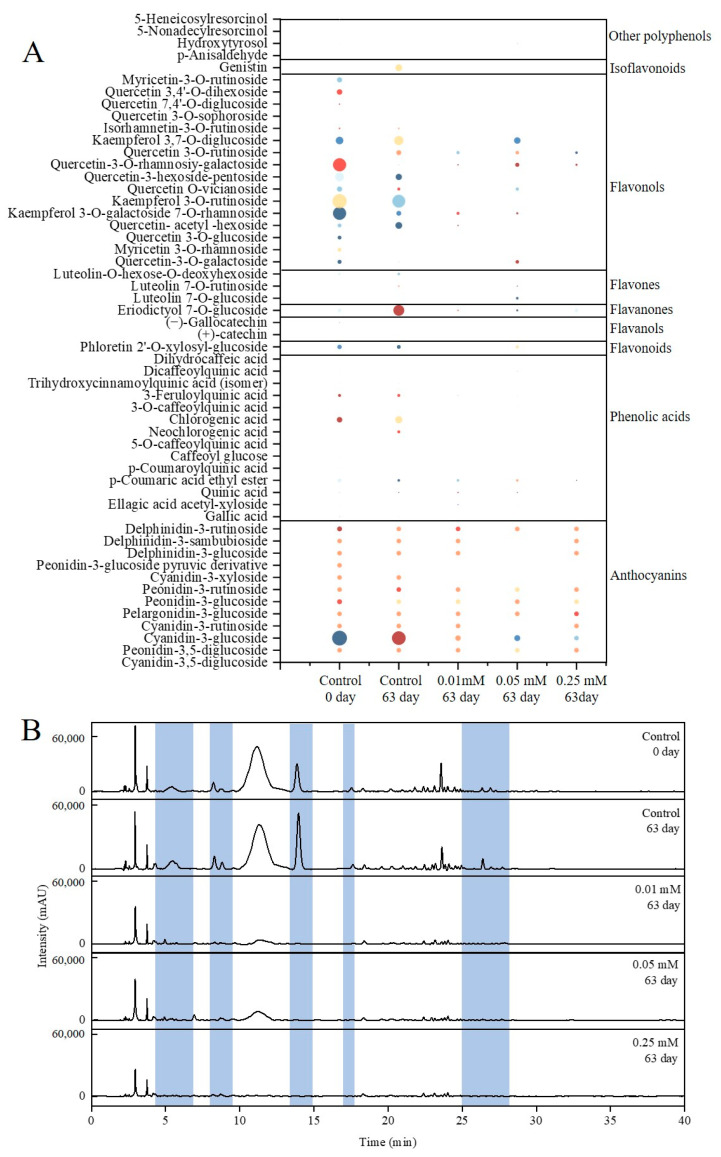
Polyphenol content (mg/100 g) (**A**) and HPLC chromatogram of polyphenols (**B**) of blue honeysuckle with melatonin treatment.

The identification of polyphenolic compounds in blue honeysuckle subjected to different melatonin treatments was accomplished through a comprehensive analysis that integrated retention times, elution order, spectral characteristics, comparison with authentic standards, and MS/MS fragmentation patterns. A total of 42 polyphenols were identified, including 12 anthocyanins, 26 non-anthocyanin phenolics, and 4 additional phenolic derivatives, as summarized in Table 1. Quantitative data for these metabolites are provided in Table A2 and Figure 4, highlighting distinct differences in polyphenol composition among treatment groups.

Anthocyanins: A total of 12 anthocyanin compounds were tentatively characterized in blue honeysuckle, predominantly comprising cyanidin, pelargonidin, delphinidin, and peonidin derivatives. Peak A1 showed a parent ion at *m/z* 611, yielding MS^2^ fragment ions at *m/z* 449 ([M − 162]^+^, corresponding to the loss of a glucose moiety) and *m/z* 287 ([M − 324]^+^, indicating the sequential loss of two glucose units), consistent with cyanidin-3,5-diglucoside. Peak A2, with a molecular ion at *m*/*z* 625, generated diagnostic fragments at *m/z* 463 ([M − 162]^+^, glucose cleavage) and *m/z* 301 (peonidin aglycone), confirming its identity as peonidin-3,5-diglucoside. Peaks A3, A5, A6, and A10 were each characterized by a neutral loss of a glucosyl residue (*m*/*z* 162), allowing their assignment as cyanidin-3-glucoside, pelargonidin-3-glucoside, peonidin-3-glucoside, and delphinidin-3-glucoside, respectively. Peaks A4, A7, and A12 displayed typical losses of a rutinoside sugar unit (*m*/*z* 308), corresponding to cyanidin-3-rutinoside, peonidin-3-rutinoside, and delphinidin-3-rutinoside, respectively. Peak A8 exhibited a molecular ion at *m/z* 419, with a fragment ion at *m/z* 287 resulting from the loss of a pentose sugar (xylose, *m/z* 132), and was thus identified as cyanidin-3-xyloside. Peak A9, possessing a parent ion at *m/z* 531, produced fragments at *m/z* 369 ([M − 162]^+^, glucose detachment) and *m/z* 301 (peonidin), attributed to peonidin-3-glucoside-pyruvic acid conjugate. Peak A11 presented a molecular ion at *m/z* 597, releasing MS^2^ fragments at *m/z* 465 ([M − 132]^+^, glucose loss) and *m/z* 303 ([M-294]^+^, corresponding to the cleavage of a sambubioside moiety), thus confirming delphinidin-3-sambubioside [2,28].

For further analysis, the non-anthocyanin polyphenols identified in blue honeysuckle treated with melatonin were classified into the following categories. Phenolic acids: Three categories of phenolic acids were characterized in blue honeysuckle, including 2 hydroxybenzoic acids, 11 hydroxycinnamic acids, and 1 hydroxyphenylpropanoic acid. Peak 9 was tentatively identified as ellagic acid acetyl-xyloside, based on its deprotonated molecular ion [M − H]^−^ at *m*/*z* 477 and key fragment ion at *m*/*z* 303. Gallic acid was assigned to peak 42, presenting a precursor ion at *m/z* 169 ([M − H]^−^). Peaks 4, 5, 6, 7, 11, 14, 15, 18, 22, 27, and 28 were annotated as cinnamoylquinic acid derivatives by comparing their MS/MS fragmentation patterns and precursor ions at *m/z* 371, 191, 367, 191, 515, 341, 353, 353, 353, 353, and 337, respectively. These correspond to trihydroxycinnamoylquinic acid isomers, p-coumaric acid ethyl ester, 3-feruloylquinic acid, quinic acid, dicaffeoylquinic acid, caffeoyl glucose, neochlorogenic acid, chlorogenic acid, 5-O-caffeoylquinic acid, 3-O-caffeoylquinic acid, and p-coumaroylquinic acid. The identification of chlorogenic acid was further confirmed by comparison with an authentic reference standard. Peak 3, attributed to dihydrocaffeic acid, was confirmed by its precursor ion [M − H]^−^ at *m*/*z* 181 and characteristic MS^2^ fragment at *m*/*z* 73 [2,29]. Dihydrochalcones: Peak 1, detected in negative ESI mode with a precursor ion at *m*/*z* 567, produced fragment ions at *m*/*z* 405 and *m/z* 273, corresponding to the sequential elimination of a glucosyl group (162 Da) and a xylosyl-glucosyl moiety (132 Da), respectively. This fragmentation pattern identified it as phloretin 2′-O-xylosyl-glucoside [30]. Flavanols: Peak 25 displayed a deprotonated molecular ion at *m/z* 289. In the MS^2^ spectra, an ion at *m/z* 245 was observed, resulting from the loss of C_2_H_4_O_2_, which identified the compound as (+)-Catechin. Peak 42 exhibited a molecular ion at *m/z* 305 and generated MS/MS fragments at *m/z* 287, resulting from the neutral loss of a water molecule (18 Da), enabling the identification of (−)-gallocatechin [29]. Flavanones: Peak 19 presented a parent ion at *m*/*z* 449, with MS/MS fragments observed at *m/z* 287 (loss of a glucosyl residue), *m*/*z* 269, and *m*/*z* 151, attributed to retro-Diels–Alder cleavage and loss of aromatic moieties. This fragmentation pattern identified the compound as eriodictyol 7-O-glucoside [31]. Flavones: Peaks 30, 35, and 37 exhibited precursor ions at *m*/*z* 447, 593, and 593 in negative ESI mode. These ions yielded major fragments at *m/z* 285, corresponding to the neutral loss of 162 Da, 308 Da, and 308 Da, respectively. Based on their fragmentation patterns, the compounds were tentatively assigned as luteolin-7-O-glucoside, luteolin-7-O-rutinoside, and luteolin-O-hexoside-O-deoxyhexoside [32]. Flavonols: Peak 13, 23, 24, 31, 32, 33, 34, 36, 39, and 41 were attributed as quercetin derivatives based on their fragmentation patterns in MS^2^ and UV spectra. These compounds were identified as quercetin 7,4′-O-diglucoside, quercetin 3-O-sophoroside, quercetin-3-O-galactoside, quercetin O-vicianoside, quercetin 3,4′-O-dihexoside, quercetin-3-hexoside-pentoside, quercetin 3-O-rutinoside, quercetin-3-O-rhamnosiy-galactoside, quercetin 3-O-glucoside, and quercetin- acetyl -hexoside, respectively. Peaks 9 and 29 share analogous MS^2^ products of *m*/*z* 315. Peak 9 was therefore established as myricetin 3-O-rhamnoside, while peak 289 is identified as myricetin-3-O-rutinoside. Peaks 10, 20, and 26 were identified as kaempferol derivatives based on data from a previous survey and their ion fragments at [M − H]^−^ with *m*/*z* 609, 593, and 593, corresponding to kaempferol 3, 7-O-diglucoside, kaempferol 3-O-galactoside 7-O-rhamnoside, and kaempferol 3-O-rutinoside. Peak 40 was characterized as isorhamnetin-3-O-rutinoside, presenting a deprotonated molecular ion [M − H]^−^ at *m*/*z* 623 and a major fragment ion at *m/z* 315, corresponding to the loss of a rutinoside moiety (*m/z* 308) [29,30]. Isoflavones: Peak 38 exhibited a precursor ion at *m*/*z* 432 in negative ionization mode, yielding a prominent fragment at *m*/*z* 269 following the cleavage of a glucosyl residue, consistent with the identity of genistin [33]. Other polyphenols: Peak 2 generated a parent ion at *m*/*z* 135 ([M − H]^−^) and product ions at *m/z* 65, facilitating its identification as p-anisaldehyde. Peak 17 was tentatively assigned as hydroxytyrosol, supported by a molecular ion at *m*/*z* 154 and diagnostic fragments at *m*/*z* 123, 109, and 95. Peak 12 exhibited a precursor ion at *m*/*z* 375 and a key fragment at *m*/*z* 191, indicative of 5-nonadecylresorcinol. Peak 21 was attributed to 5-heneicosylresorcinol, with [M − H]^−^ at *m*/*z* 403 and product ions observed at *m*/*z* 223, 179, and 125 [34,35,36].

### 3.6. Quantitative Analysis of Polyphenols of Blue Honeysuckle Fruits

Polyphenol content is a key indicator of the nutraceutical value of blue honeysuckle berries [2]. The major polyphenolic compounds identified in fresh fruits included cyanidin-3-glucoside, chlorogenic acid, phloretin 2′-O-xylosyl-glucoside, and kaempferol 3-O-rutinoside. During storage, anthocyanin profiles exhibited notable changes: five anthocyanins increased, while seven declined over 63 d. Peonidin-3-glucoside pyruvic derivative became undetectable, likely due to its high susceptibility to degradation or transformation under storage conditions [37]. Melatonin treatment significantly affected polyphenol dynamics [3]. After 63 d, cyanidin-3-glucoside levels in melatonin-treated fruits were 4–6-times lower than in the control. In untreated samples, storage led to increases of 39.92, 196.43, 12.94, and 100.62 mg/100 g in phenolic acids, flavanones, flavones, and isoflavonoids, respectively, likely due to cold-induced stimulation of phenolic biosynthesis during postharvest handling [37]. Conversely, melatonin application suppressed the overall accumulation of phenolic acids and flavonoids. However, specific compounds such as p-coumaroylquinic acid, caffeoyl glucose, 5-O-caffeoylquinic acid, 3-O-caffeoylquinic acid, luteolin-7-O-glucoside, and hydroxytyrosol were elevated in fruits treated with 0.05 mM melatonin. Overall, melatonin treatment reduced the levels of anthocyanins, phenolic acids, flavonoids, flavanols, flavanones, flavones, flavonols, isoflavonoids, and other polyphenols in blue honeysuckle. This trend contrasts with some previous studies that reported enhanced polyphenol accumulation following melatonin application [7,8]. One possible explanation is that melatonin may modify the tissue redox status, thereby indirectly influencing polyphenol stability and content [38].

### 3.7. Antioxidant Capacity of Blue Honeysuckle Fruits

Reactive oxygen species (ROS) play a critical role in regulating fruit senescence; however, their excessive accumulation accelerates postharvest quality deterioration. Exogenous melatonin has been shown to enhance ROS scavenging capacity by modulating non-enzymatic antioxidant defenses during cold storage of harvested fruits [3]. In this study, three antioxidant assays were employed to evaluate the effects of melatonin on the oxidative stability of blue honeysuckle during refrigerated storage (Table A1 and Figure 3). Over time, both treated and untreated fruits showed a progressive increase in DPPH radical scavenging activity. By the end of storage, melatonin-treated samples exhibited significantly higher DPPH scavenging capacity than the control (*p* < 0.05). A similar trend was observed for ABTS^+^·radical scavenging activity. After 35 d, melatonin-treated fruits displayed markedly enhanced ABTS scavenging capacity, with the 0.25 mM treatment yielding the highest value at the end of storage (15.78 mg Trolox/g FW, *p* < 0.05). In contrast, FRAP-reducing power gradually declined throughout storage. Nevertheless, after 63 d, fruits treated with 0.01 mM and 0.25 mM melatonin maintained significantly higher FRAP values compared to the control. These findings align with previous reports indicating that 1 mM melatonin significantly increased DPPH and ABTS values in strawberries [8]. Although polyphenols are recognized as non-enzymatic antioxidants, this study found no positive correlation between total phenolic content and antioxidant capacity, a result consistent with observations in mango [39]. This suggests that the enhanced antioxidant performance of melatonin-treated fruits may be mediated by enzymatic antioxidant pathways [3]. Additionally, melatonin-treated fruits initially exhibited slightly elevated MDA levels compared to the control during early storage. However, as storage progressed, melatonin significantly suppressed MDA accumulation. By day 63, MDA levels were reduced by 85.00%, 47.17%, and 97.23% in fruits treated with 0.01 mM, 0.05 mM, and 0.25 mM melatonin, respectively, relative to the control. These results demonstrate that melatonin effectively mitigates oxidative damage and delays postharvest senescence in blue honeysuckle. Similar effects were reported by Liu et al., who observed a 45.48% reduction in MDA content in strawberries treated with 0.1 mM melatonin [8].

### 3.8. Multivariate Statistical Analysis

**Figure 5:**
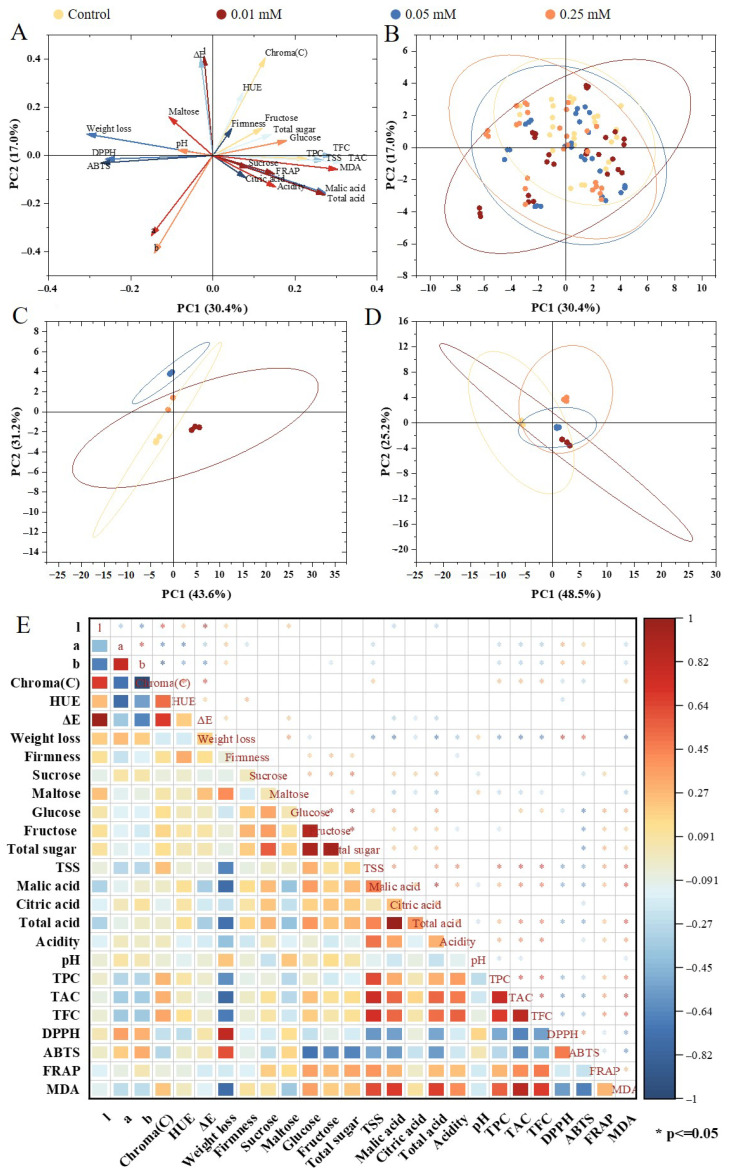
Multivariate analysis of postharvest blue honeysuckle. PCA plot illustrating associations among postharvest blue honeysuckle indexes: PC1 (x) vs. PC2 (y) (**A**). Principal component analysis (PCA) plot showing associations of blue honeysuckle with melatonin (0.01, 0.05, 0.25 mM) treatment (**B**). Principal component analysis (PCA) plot at 0 d (**C**). Principal component analysis (PCA) plot at 63 d (**D**). Pearson’s correlation coefficient among postharvest blue honeysuckle indexes at *p* ≤ 0.05 (**E**).

As shown in the PCA loading plot (Figure 5A), firmness, fructose, and glucose were primarily located in the first quadrant, indicating strong positive correlations among these variables. Their positioning suggests that they contribute substantially to the total variance and are key indicators of postharvest quality in blue honeysuckle. In contrast, color parameters such as a and b clustered in the third quadrant, displaying a clear negative correlation with the quality attributes. This pattern likely reflects quality deterioration during storage, potentially linked to anthocyanin degradation or browning, as evidenced by color changes [40]. The dataset was reduced into two principal components, which together explained 47.4% of the total variance. Specifically, PC1 accounted for 30.4%, and PC2 explained 17.0% (Figure 5B). Following melatonin treatment, at day 0, PC1 and PC2 explained 43.6% and 31.2% of the variance, respectively, yielding a cumulative explanation of 74.8% (Figure 5C). By day 63, the variance explained by PC1 increased to 48.5%, while PC2 decreased to 25.2%, totaling 73.7% (Figure 5D). This temporal shift suggests that PC1-associated variables increasingly dominated the variability among samples as storage progressed. To further explore interrelationships among postharvest quality parameters, Pearson’s correlation analysis was conducted (Figure 5E, Table A3). Weight loss exhibited a strong positive correlation with DPPH activity (r = 0.81), indicating that increased oxidative stress may contribute to moisture depletion during storage. A moderate positive correlation (r = 0.48) was found between TFC and FRAP values, suggesting that flavonoids play a meaningful role in enhancing antioxidant potential. Conversely, a strong negative correlation was observed between ABTS activity and glucose content (r = −0.71), implying that simple sugar accumulation may be inversely associated with antioxidant capacity, potentially due to metabolic shifts during senescence or the degradation of polyphenol compounds. These results indicate that postharvest quality traits in blue honeysuckle are tightly interconnected, and melatonin treatment may help to preserve desirable attributes by enhancing antioxidant defenses and delaying textural degradation. While these findings align with previous reports showing that melatonin mitigates oxidative stress and reduces weight loss during cold storage [41], our study also revealed a reduction in polyphenol levels, contrary to studies that reported melatonin-induced increases in total phenolics. This discrepancy may reflect species-specific responses and could be influenced by factors such as fruit maturity, storage duration, and initial antioxidant status [7].

### 3.9. Regulatory Mechanisms of Melatonin on Sugars, Acids, and Polyphenols

**Figure 6:**
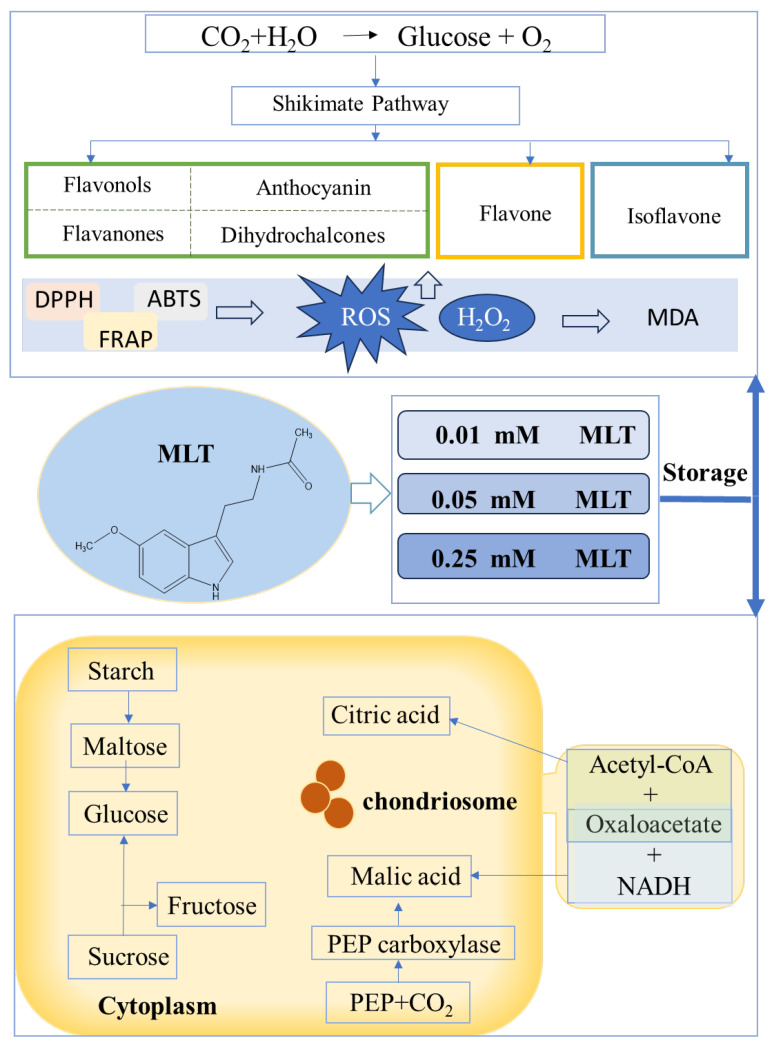
Illustration of the potential mechanism and changes in metabolite (polyphenols, sugars, and acids) and antioxidant capacity behind the melatonin treatment.

Melatonin treatment plays a multifaceted role in preserving fruit quality during cold storage by modulating both oxidative and metabolic pathways (Figure 6). During postharvest storage, blue honeysuckle berries typically exhibit increased oxidative stress, characterized by elevated ROS, including H_2_O_2_ and lipid peroxidation, as indicated by rising MDA levels. These oxidative changes are closely linked to declines in firmness and nutritional quality. However, exogenous melatonin application significantly mitigated these effects, which may be associated with enhanced antioxidant capacity, as indicated by improved DPPH, ABTS, and FRAP activities. Melatonin is a pleiotropic molecule with diverse roles in plant physiology. It acts primarily as a free radical scavenger, directly neutralizing ROS and RNS, and regulates the expression and activity of antioxidant enzymes (e.g., SOD, CAT, APX). Moreover, its recent designation as a plant hormone or “master regulator” reflects its involvement in stress signaling, growth, and senescence pathways [8,9]. In addition to its antioxidant effects, melatonin also influenced carbohydrate metabolism [42]. In the cytoplasm, starch is enzymatically degraded into maltose, glucose, and subsequently fructose and sucrose—sugars essential for maintaining osmotic balance, flavor, and stress resistance. Our results showed that 0.01 mM and 0.05 mM melatonin treatments stabilized fructose and glucose accumulation, helping to preserve sugar levels throughout extended storage. Similar outcomes have been reported in blueberries, where melatonin delayed senescence by modulating carbohydrate metabolism [40]. Moreover, sugar metabolism intermediates serve as substrates for the tricarboxylic acid (TCA) cycle in mitochondria. Compounds such as phosphoenolpyruvate, oxaloacetate, malic acid, and citric acid are central to energy production and cellular homeostasis [43]. By promoting these energy-generating pathways, melatonin may support sustained metabolic activity and delay cellular degradation. Overall, melatonin acts not only as a potent antioxidant but also as a key regulator of sugar metabolism, ultimately enhancing fruit quality and extending postharvest storage life.

## 4. Conclusions

Melatonin treatment preserved postharvest firmness of blue honeysuckle in a concentration-dependent manner, with 0.05 mM showing the greatest effect. This treatment enhanced sugars (sucrose, maltose, glucose, fructose) and malic acid, improving the sugar–acid balance and flavor retention. Total phenolics, anthocyanins, and flavonoids increased during early storage but declined thereafter. These changes were accompanied by enhanced antioxidant capacity, particularly during early storage, as measured by DPPH, ABTS, and FRAP assays. HPLC-ESI-QTOF-MS^2^ analysis revealed dynamic changes in phenolic profiles. While total phenolic acids and flavonoids decreased under melatonin treatment, several specific compounds, including p-coumaroylquinic acid, caffeoyl glucose, and hydroxytyrosol, were relatively elevated, which may be associated with improved fruit quality. Overall, 0.05 mM melatonin effectively improved storability and maintained blue honeysuckle quality during cold storage. Melatonin may serve as a promising postharvest treatment for improving the shelf life, texture, and nutritional value of blue honeysuckle fruit during commercial cold storage. Future research should aim to further elucidate the molecular mechanisms by which melatonin regulates postharvest quality in blue honeysuckle fruits. Transcriptomic analysis may help to clarify gene expression changes related to polyphenol biosynthesis, sugar–acid metabolism, and antioxidant defense pathways. Moreover, sensory evaluation using trained panels and consumer testing will be valuable for assessing the effects of melatonin treatment on flavor, texture, and overall acceptability, thereby linking biochemical alterations with consumer perception.

## Figures and Tables

**Table 1:** Characterization of polyphenol compounds of blue honeysuckle.

Peak	Retention Time (min)	Formula	Molecular Weight (MW)	MS Expected Value [M]^+^/([M − H]^−^) (*m*/*z*)	MS Found Value ([M]^+^/([M − H]^−^))(*m*/*z*)	MS^2^ (*m*/*z*)	Error (ppm)	Tentative Assignment	HPLC-DAD λ Max (nm)
Anthocyanins									
A1	5.57	C_27_H_31_O_16_^+^	611	611.1609	611.1617	449.1095/287.0555	1.31	Cyanidin-3,5-diglucoside	522,285
A2	7.9	C_28_H_33_O_16_^+^	625	625.1765	625.1771	301.0706/463.1242/625.1799	0.96	Peonidin-3,5-diglucoside	515
A3	11.42	C_21_H_21_O_11_^+^	449	449.1077	449.1076	287.0545	−0.22	Cyanidin-3-glucoside	516,280
A4	12.19	C_27_H_31_O_15_^+^	595	595.1654	595.166	287.0554/595.1626	1.01	Cyanidin-3-rutinoside	517
A5	14.69	C_21_H_21_O_10_^+^	433	433.1131	433.1129	271.0607	−0.46	Pelargonidin-3-glucoside	508,273
A6	16.76	C_22_H_23_O_11_^+^	463	463.1234	463.1238	301.0703, 286.0455	0.86	Peonidin-3-glucoside	513,278
A7	17.98	C_28_H_33_O_15_^+^	609	609.1813	609.1815	301.0708/609.1806	0.33	Peonidin-3-rutinoside	517
A8	18.65	C_20_H_19_O_10_^+^	419	419.0976	419.0981	287.0543	1.19	Cyanidin-3-xyloside	524
A9	18.73	C_23_H_31_O_14_^+^	531	531.2042	531.2042	301.1443/369.1039	0	Peonidin-3-glucoside pyruvic derivative	527,246
A10	20.93	C_21_H_21_O_12_^+^	465	465.1731	465.1729	303.1207/465.1716	−0.43	Delphinidin-3-glucoside	520,279
A11	22.63	C_26_H_29_O_14_^+^	597	597.1451	597.1456	303.0512/465.0996	0.84	Delphinidin-3-sambubioside	527
A12	23.52	C_27_H_31_O_16_^+^	611	611.2808	611.2813	303.0504	0.82	Delphinidin-3-rutinoside	550,261
Phenolic acids									
Hydroxybenzoic acids									
9	4.13	C_21_H_16_O_13_	478	477.9508	477.9531	477.9506/303.8766	4.81	Ellagic acid acetyl-xyloside	259,281
42	7.89	C_7_H_6_O_5_	170	169.0145	169.0149	169.0108/151.0021	2.37	Gallic acid	284
Hydroxycinnamic acids									
4	2.65	C_16_H_20_O_10_	372	371.0473	371.048	191.05	1.89	Trihydroxycinnamoylquinic acid (isomer)	252
5	2.73	C_11_H_12_O_3_	192	191.0558	191.0562	87.0122/111.0099/85.0330/129.0197	2.09	p-Coumaric acid ethyl ester	287
6	2.95	C_17_H_20_O_9_	368	367.0674	367.068	367.0725/191.0509/135.0447	1.63	3-Feruloylquinic acid	251
7	3.74	C_7_H_12_O_6_	192	191.0201	191.0204	191.0497/173.0418	1.57	Quinic acid	242,277
11	5.13	C_25_H_24_O_12_	516	515.1409	515.1425	515.0887/323.0542/191.0507	3.11	Dicaffeoylquinic acid	282
14	7.14	C_15_H_18_O_9_	342	341.0873	341.0893	341.0633/191.0520/179.0305	5.86	Caffeoyl glucose	278
15	7.72	C_16_H_18_O_9_	354	353.0878	353.0887	353.0632/191.0502/179.0297	2.55	Neochlorogenic acid	287
18	12.78	C_16_H_18_O_9_	354	353.0871	353.0886	191.0499	4.25	Chlorogenic acid	281
22	17.40	C_16_H_18_O_9_	354	353.0869	353.0885	191.0497	4.53	5-O-caffeoylquinic acid	294
27	19.23	C_16_H_18_O_9_	354	353.0718	353.0733	191.0499	4.25	3-O-caffeoylquinic acid	234,285
28	19.44	C_16_H_18_O_8_	338	337.0921	337.0937	191.0506/163.0376	4.75	p-Coumaroylquinic acid	233,283
Hydroxyphenylpropanoic acids									
3	2.55	C_9_H_10_O_4_	182	181.0566	181.0564	181.0664/73.0340/59.0189	−1.1	Dihydrocaffeic acid	243,283
Flavonoids									
Dihydrochalcones									
1	2.26	C_26_H_32_O_14_	568	567.1907	567.195	567.1353/227.0822/191.0507	7.58	Phloretin 2′-O-xylosyl-glucoside	252
Flavanols									
25	18.67	C_15_H_14_O_6_	290	289.0715	289.0727	245.0545	4.15	(+)-Catechin	242,275
42	26.27	C_15_H_14_O_7_	306	305.0667	305.068	305.0514/287.0401	4.26	(-)-Gallocatechin	262
Flavanones									
19	16.11318602	C_21_H_22_O_11_	450	449.1069	449.1091	449.0677/287.0401/269.0300/151.0019	4.9	Eriodictyol 7-O-glucoside	278
Flavones									
30	20.66	C_21_H_20_O_11_	448	447.0922	447.0938	285.0224	3.58	Luteolin 7-O-glucoside	252
35	22.72	C_27_H_30_O_16_	594	593.1533	593.153	593.0891/285.0233	−0.51	Luteolin 7-O-rutinoside	261
37	23.29	C_26_H_28_O_16_	594	593.1501	593.1516	593.0874/285.0220/284.0148	2.53	Luteolin-O-hexose-O-deoxyhexoside	254
Flavonols									
9	4.27	C_21_H_20_O_12_	464	463.1081	463.1106	315.0501/153.0176/129.0188	5.4	Myricetin 3-O-rhamnoside	255
10	4.58	C_27_H_30_O_16_	610	609.145	609.1487	609.0844/447.0542/285.0231	6.07	Kaempferol 3,7-O-diglucoside	275
13	7.12	C_27_H_30_O_17_	626	625.1403	625.1424	625.0735/300.0073/301.0150/271.0087	3.36	Quercetin 7,4′-O-diglucoside	245,283
20	16.68	C_27_H_30_O_15_	594	593.271	593.2746	593.0872/285.0216	6.07	Kaempferol 3-O-galactoside 7-O-rhamnoside	254
23	18.32	C_27_H_30_O_17_	626	625.1202	625.12	625.0721/301.0151/300.0079	−0.32	Quercetin 3-O-sophoroside	242,276
24	18.35	C_27_H_30_O_17_	464	463.0858	463.0887	463.0450/301.0157/300.0071	6.26	Quercetin-3-O-galactoside	259
26	19.08	C_27_H_30_O_15_	594	594.1203	594.1231	593.0872/285.0228/284.0157	4.71	Kaempferol 3-O-rutinoside	273
29	20.20	C_27_H_30_O_17_	626	625.1786	625.1795	625.0753/301.0155/300.0074	1.44	Myricetin-3-O-rutinoside	261
31	21.12	C_26_H_28_O_16_	596	595.1283	595.1316	595.0653/301.0152	5.55	Quercetin O-vicianoside	249
32	21.88	C_24_H_34_O_19_	626	625.1597	625.1632	625.0735/301.0150	5.6	Quercetin 3,4′-O-dihexoside	273
33	22.03	C_26_H_32_O_18_	596	595.2028	595.2052	301.0144/300.0062	4.03	Quercetin-3-hexoside-Pentoside	282
34	22.44	C_27_H_30_O_16_	610	610.1485	610.1477	595.0679/301.0163/300.0086	−1.31	Quercetin 3-O-rutinoside	277
36	22.94	C_27_H_30_O_16_	610	609.144	609.1472	301.0146/300.0063	5.25	Quercetin-3-O-rhamnosiy-galactoside	243,275
39	23.97	C_21_H_20_O_12_	464	463.2171	463.2183	463.0450/301.0157/300.0071	2.59	Quercetin 3-O-glucoside	279
40	24.36	C_28_H_32_O_16_	624	623.1589	623.1625	315.0291/314.0215/300.0074	5.78	Isorhamnetin-3-O-rutinoside	241,273
41	25.65	C_23_H_25_O_17_	506	505.0983	505.0992	505.0482/301.0155/300.0071	1.78	Quercetin- acetyl-hexoside	250
Isoflavonoids									
38	23.57	C_21_H_20_O_10_	432	431.0981	431.0991	431.0597/269.0302/268.0221	2.32	Genistin	263
Other polyphenols									
Hydroxybenzaldehydes									
2	2.32	C_8_H_8_O_2_	135	134.0475	134.0476	134.0463/107.0380	0.75	p-Anisaldehyde	235
Tyrosols									
17	8.51	C_8_H_10_O_3_	154	154.0624	154.0622	109.0321	−1.3	Hydroxytyrosol	270
Alkylphenols									
12	7.04	C_16_H_24_O_10_	376	375.2917	375.2932	191.0649	4	5-Nonadecylresorcinol	268, 297
21	17.07	C_27_H_48_O_2_	404	403.1591	403.1577	403.1261/223.0879/179.1023/125.0625	−3.47	5-Heneicosylresorcinol	237

## Data Availability

The original contributions presented in this study are included in the article. Further inquiries can be directed to the corresponding author.

## Appendix A

**Table A1:** Overview of postharvest quality of blue honeysuckle.

Treatment	0 Day	3 Day	7 Day	14 Day	21 Day	28 Day	35 Day	49 Day	63 Day
l									
control	26.39 ± 0 ^Bd^	32.53 ± 2.62 ^Aabc^	30.47 ± 2.46 ^Ac^	33.69 ± 0.45 ^Aab^	32.73 ± 2.15 ^Babc^	30.89 ± 0.96 ^Bbc^	35.41 ± 1.42 ^Aa^	35.04 ± 1.67 ^Aa^	33.81 ± 1.45 ^Aab^
0.01 mM	33.24 ± 0.85 ^Ab^	29.64 ± 1.02 ^ABcd^	30.19 ± 4.19 ^Abcd^	28.66 ± 1.24 ^Bcd^	37.96 ± 0.39 ^Aa^	29.09 ± 1.44 ^Bcd^	27.37 ± 1.37 ^Bd^	31.65 ± 0.52 ^Bbc^	28.47 ± 1.34 ^Bcd^
0.05 mM	27.69 ± 0.3 ^Bcd^	30.63 ± 0.49 ^ABabc^	31.1 ± 5.14 ^Aabc^	34.14 ± 2.52 ^Aa^	31.22 ± 0.36 ^Babc^	34.32 ± 1.12 ^Aa^	26.46 ± 0.73 ^Bd^	32.15 ± 0.34 ^Bab^	29.21 ± 0.3 ^Bbcd^
0.25 mM	26.32 ± 2.49 ^Bc^	28.65 ± 1.28 ^Bc^	32.53 ± 2.98 ^Ab^	27.38 ± 2.91 ^Bc^	32 ± 0.6 ^Bb^	33.49 ± 1.77 ^Aab^	36.27 ± 1.28 ^Aa^	34.31 ± 0.83 ^Aab^	34.49 ± 0.68 ^Aab^
a									
control	−0.47 ± 0.02 ^Aa^	−1.05 ± 0.05 ^Ccd^	−1.07 ± 0.07 ^Bcd^	−1.06 ± 0.04 ^Ccd^	−0.98 ± 0.06 ^Bcbd^	−0.88 ± 0.1 ^Bbc^	−0.83 ± 0.07 ^Cb^	−1.09 ± 0.1 ^Dde^	−1.25 ± 0.22 ^Ce^
0.01 mM	−1.05 ± 0.13 ^Ce^	−0.64 ± 0.03 ^Bcd^	−1.06 ± 0.05 ^Be^	−0.77 ± 0.05 ^Bd^	−1.28 ± 0.09 ^Cf^	1.05 ± 0.05 ^Ab^	−0.59 ± 0.04 ^Bc^	−0.59 ± 0.02 ^Ac^	2.42 ± 0.23 ^Aa^
0.05 mM	−0.71 ± 0.08 ^Be^	−0.49 ± 0.04 ^Ac^	0.38 ± 0.05 ^Aa^	−1.33 ± 0.03 ^Dh^	−0.99 ± 0.04 ^Bfg^	−1.05 ± 0.02 ^Cg^	0.18 ± 0.02 ^Ab^	−0.95 ± 0.03 ^Cf^	−0.57 ± 0.03 ^Bd^
0.25 mM	−0.54 ± 0.03 ^Ab^	−0.42 ± 0.04 ^Aa^	−1.11 ± 0.05 ^Be^	−0.43 ± 0.02 ^Aa^	−0.72 ± 0.04 ^Ac^	−1.11 ± 0.07 ^Ce^	−0.87 ± 0.06 ^Cd^	−0.73 ± 0.03 ^Bc^	−0.47 ± 0.02 ^Bab^
b									
control	−3.7 ± 0.34 ^Ba^	−6.81 ± 0.73 ^Cc^	−5.2 ± 0.17 ^Bb^	−6.31 ± 0.21 ^Cc^	−5.58 ± 0.19 ^Cb^	−5.48 ± 0.24 ^Bb^	−6.53 ± 0.39 ^Dc^	−5.33 ± 0.33 ^Bb^	−6.39 ± 0.21 ^Cc^
0.01 mM	−5.57 ± 0.69 ^Ad^	−4.42 ± 0.55 ^ABc^	−5.3 ± 0.37 ^Bd^	−5.44 ± 0.24 ^Bd^	−6.75 ± 0.32 ^De^	−2.44 ± 0.16 ^Ab^	−4.12 ± 0.04 ^Bc^	−4.36 ± 0.13 ^Ac^	−1.52 ± 0.19 ^Aa^
0.05 mM	−4.32 ± 0.24 ^Bbc^	−5.04 ± 0.24 ^Bde^	−4.37 ± 0.48 ^Abc^	−6.5 ± 0.3 ^Cf^	−4.84 ± 0.21 ^Bcd^	−6.32 ± 0.38 ^Cf^	−2.61 ± 0.25 ^Aa^	−5.45 ± 0.12 ^Be^	−4 ± 0.42 ^Bb^
0.25 mM	−3.52 ± 0.14 ^Bab^	−3.9 ± 0.12 ^Aabc^	−6.18 ± 0.18 ^Cg^	−3.18 ± 0.8 ^Aa^	−4.26 ± 0.34 ^Abcd^	−5.77 ± 0.48 ^BCfg^	−5.39 ± 0.26 ^Cef^	−4.99 ± 0.66 ^ABde^	−4.34 ± 0.26 ^Bcd^
Chroma (c)									
control	3.73 ± 0.34 ^BCc^	6.89 ± 0.72 ^Aa^	5.31 ± 0.16 ^Bb^	6.4 ± 0.2 ^Aa^	5.66 ± 0.19 ^Bb^	5.55 ± 0.22 ^Bb^	6.58 ± 0.38 ^Aa^	5.44 ± 0.31 ^Ab^	6.52 ± 0.24 ^Aa^
0.01 mM	5.67 ± 0.7 ^Ab^	4.47 ± 0.55 ^BCc^	5.41 ± 0.37 ^Bb^	5.49 ± 0.23 ^Bb^	6.87 ± 0.3 ^Aa^	2.66 ± 0.17 ^Cd^	4.16 ± 0.04 ^Cc^	4.4 ± 0.12 ^Bcd^	2.86 ± 0.16 ^Cd^
0.05 mM	4.38 ± 0.23 ^Bd^	5.06 ± 0.24 ^Bbc^	4.39 ± 0.48 ^Cd^	6.63 ± 0.29 ^Aa^	4.94 ± 0.2 ^Cc^	6.41 ± 0.37 ^Aa^	2.62 ± 0.25 ^De^	5.53 ± 0.12 ^Ab^	4.04 ± 0.41 ^Bd^
0.25 mM	3.56 ± 0.14 ^Cf^	3.93 ± 0.12 ^Cef^	6.28 ± 0.19 ^Aa^	3.21 ± 0.79 ^Cd^	4.32 ± 0.34 ^Dde^	5.88 ± 0.48 ^ABab^	5.46 ± 0.24 ^Bbc^	5.04 ± 0.65 ^ABcd^	4.37 ± 0.26 ^Bde^
Hue (h)									
control	262.68 ± 0.37 ^Aa^	261.17 ± 1.02 ^Bab^	258.4 ± 0.84 ^Bd^	260.45 ± 0.66 ^ABbcd^	260.07 ± 0.28 ^Abcd^	260.81 ± 1.41 ^Abc^	262.74 ± 1.02 ^Aa^	258.39 ± 1.61 ^Cd^	258.94 ± 1.62 ^Acd^
0.01 mM	259.29 ± 0.16 ^Cab^	261.74 ± 0.91 ^Bab^	258.63 ± 0.7 ^ABb^	261.92 ± 0.74 ^Aab^	259.2 ± 1.12 ^ABab^	113.27 ± 0.34 ^Bd^	261.89 ± 0.58 ^ABab^	262.33 ± 0.49 ^Aa^	147.68 ± 4.89 ^Bc^
0.05 mM	260.67 ± 1.41 ^BCbc^	264.41 ± 0.32 ^Aa^	95.06 ± 0.9 ^Ce^	258.44 ± 0.62 ^Bd^	258.45 ± 0.8 ^Bd^	260.52 ± 0.64 ^Ac^	93.89 ± 0.63 ^Ce^	260.15 ± 0.28 ^BCbc^	261.8 ± 0.67 ^Ab^
0.25 mM	261.24 ± 0.14 ^Bbc^	263.84 ± 0.76 ^Aa^	259.85 ± 0.39 ^Acd^	262.05 ± 1.82 ^Ab^	260.4 ± 0.43 ^Abcd^	259.11 ± 0.86 ^Acd^	260.76 ± 1.08 ^Bbc^	261.56 ± 0.84 ^ABb^	263.82 ± 0.28 ^Aa^
ΔE									
control	0.25 ± 0.15 ^Cd^	7 ± 2.37 ^Abc^	4.44 ± 2.3 ^Ac^	7.78 ± 0.46 ^Ab^	6.64 ± 2.09 ^Babc^	4.87 ± 0.88 ^Bbc^	9.48 ± 1.27 ^Aa^	8.83 ± 1.67 ^Aa^	7.94 ± 1.44 ^Aa^
0.01 mM	7.13 ± 0.99 ^Ab^	3.38 ± 0.94 ^ABcd^	4.87 ± 2.85 ^Ac^	2.99 ± 0.78 ^Bcd^	11.99 ± 0.33 ^Aa^	3.41 ± 1.2 ^Bcd^	1.26 ± 1.1 ^Bd^	5.31 ± 0.51 ^Bbc^	4.29 ± 0.65 ^Bc^
0.05 mM	1.49 ± 0.16 ^BCc^	4.45 ± 0.51 ^ABbc^	5.24 ± 4.54 ^Aab^	8.3 ± 2.43 ^Aa^	5 ± 0.33 ^Bb^	8.38 ± 1.09 ^Aa^	1.41 ± 0.11 ^Bc^	6.04 ± 0.34 ^Bab^	2.86 ± 0.3 ^Bbc^
0.25 mM	1.88 ± 0.99 ^Bc^	2.29 ± 1.25 ^Bc^	6.73 ± 2.69 ^Ab^	2.68 ± 0.45 ^Bc^	5.65 ± 0.62 ^Bb^	7.43 ± 1.8 ^Ab^	10.04 ± 1.25 ^Aa^	8.04 ± 0.92 ^Aab^	8.13 ± 0.67 ^Aab^
Weight loss (%)									
control	0.00% ± 0.00 ^Ag^	0.05% ± 0.00 ^Cg^	0.08% ± 0.00 ^Bg^	0.69% ± 0.00 ^Cf^	1.11% ± 0.00 ^Ce^	1.49% ± 0.00 ^Bd^	2.14% ± 0.00 ^Cc^	2.91% ± 0.00 ^Cb^	3.66% ± 0.00 ^Ca^
0.01 mM	0.00% ± 0.00 ^Ai^	0.47% ± 0.00 ^Bh^	1.25% ± 0.00 ^Ag^	2.12% ± 0.00 ^Bf^	2.75% ± 0.00 ^Be^	3.21% ± 0.00 ^Ad^	3.74% ± 0.00 ^Bc^	4.65% ± 0.00 ^Bb^	5.47% ± 0.00 ^Ba^
0.05 mM	0.00% ± 0.00 ^Ai^	0.82% ± 0.00 ^Ah^	1.58% ± 0.00 ^Ag^	2.59% ± 0.00 ^Af^	3.27% ± 0.00 ^Ae^	3.74% ± 0.00 ^Ad^	4.35% ± 0.00 ^Ac^	5.37% ± 0.00 ^Ab^	6.30% ± 0.01 ^Aa^
0.25 mM	0.00% ± 0.00 ^Ah^	0.74% ± 0.00 ^Ag^	1.58% ± 0.00 ^Af^	2.59% ± 0.00 ^Ae^	3.41% ± 0.00 ^Ad^	3.64% ± 0.00 ^Ad^	4.17% ± 0.00 ^ABc^	5.51% ± 0.00 ^Ab^	6.44% ± 0.00 ^Aa^
Firmness (N)									
control	1.31 ± 0.13 ^Aab^	1.57 ± 0.52 ^Aa^	1.32 ± 0.8 ^Aab^	1.35 ± 0.15 ^Aab^	0.90 ± 0.25 ^BCb^	0.88 ± 0.13 ^Bb^	1.35 ± 0.13 ^Aab^	1.23 ± 0.13 ^Aab^	0.73 ± 0.02 ^Cb^
0.01 mM	1.04 ± 0.15 ^Aabcd^	0.99 ± 0.2 ^Abcd^	1.32 ± 0.37 ^Aa^	0.83 ± 0.1 ^Ccd^	1.15 ± 0.14 ^Babc^	0.87 ± 0.16 ^Bcd^	1.02 ± 0.13 ^Aabcd^	1.22 ± 0.05 ^Aab^	0.76 ± 0.03 ^Cd^
0.05 mM	1.08 ± 0.13 ^Abc^	1.3 ± 0.25 ^Aa^	0.85 ± 0.05 ^Ad^	0.88 ± 0.03 ^Cd^	0.77 ± 0.03 ^Cd^	0.92 ± 0.05 ^Bcd^	0.83 ± 0.05 ^Ad^	1.19 ± 0.07 ^Aab^	1.25 ± 0.03 ^Bab^
0.25 mM	1.2 ± 0.39 ^Aabc^	1.22 ± 0.25 ^Aabc^	0.93 ± 0.21 ^Ac^	1.1 ± 0.12 ^Babc^	1.57 ± 0.24 ^Aa^	1.52 ± 0.28 ^Aab^	0.88 ± 0.22 ^Ac^	1.19 ± 0.23 ^Aabc^	1.55 ± 0.02 ^Aab^
Sucrose (%)									
control	0.28 ± 0 ^Ch^	1.04 ± 0.01 ^Dc^	1.01 ± 0.01 ^Ad^	1.19 ± 0 ^Aa^	0.75 ± 0.01 ^Cf^	1.03 ± 0.01 ^Bc^	1.12 ± 0 ^Cb^	0.86 ± 0 ^Ae^	0.67 ± 0 ^Dg^
0.01 mM	0.18 ± 0.01 ^Dh^	1.82 ± 0 ^Aa^	0.83 ± 0.02 ^Cg^	1.12 ± 0 ^Bc^	1.01 ± 0.01 ^Be^	0.97 ± 0.01 ^Cf^	1.2 ± 0 ^Ab^	0.81 ± 0.02 ^Bg^	1.04 ± 0.01 ^Be^
0.05 mM	1.48 ± 0.01 ^Aa^	1.23 ± 0 ^Cb^	0.97 ± 0 ^Bg^	0.98 ± 0 ^Cf^	1 ± 0.01 ^Be^	1.06 ± 0 ^Ad^	1.1 ± 0.01 ^Dc^	0.77 ± 0 ^Ci^	0.86 ± 0 ^Ch^
0.25 mM	1.29 ± 0.01 ^Ba^	1.28 ± 0.01 ^Bb^	0.97 ± 0.01 ^Bg^	0.58 ± 0 ^Di^	1.15 ± 0 ^Ad^	1.02 ± 0.01 ^Bf^	1.18 ± 0 ^Bc^	0.8 ± 0 ^Bh^	1.06 ± 0.01 ^Ae^
Maltose (%)									
control	0.13 ± 0 ^Dg^	0.05 ± 0 ^Dh^	0.2 ± 0.01 ^Bf^	0.48 ± 0.01 ^Bc^	0.34 ± 0 ^Cd^	0.53 ± 0.01 ^Ab^	0.58 ± 0 ^Ba^	0.52 ± 0 ^Bb^	0.31 ± 0.01 ^BCe^
0.01 mM	0.54 ± 0 ^Ab^	0.26 ± 0 ^Af^	0.12 ± 0.01 ^Cg^	0.48 ± 0 ^Bd^	0.5 ± 0.01 ^Ac^	0.5 ± 0.01 ^Cc^	0.61 ± 0 ^Aa^	0.55 ± 0.01 ^Ab^	0.3 ± 0 ^Ce^
0.05 mM	0.41 ± 0.01 ^Ce^	0.23 ± 0 ^Bf^	0.2 ± 0.01 ^Bg^	0.51 ± 0 ^Ab^	0.47 ± 0 ^Bc^	0.51 ± 0 ^Bb^	0.56 ± 0.01 ^Ca^	0.47 ± 0.02 ^Cc^	0.43 ± 0 ^Ad^
0.25 mM	0.46 ± 0.01 ^Be^	0.2 ± 0.01 ^Ch^	0.36 ± 0.01 ^Af^	0.31 ± 0.01 ^Cg^	0.47 ± 0.01 ^Bd^	0.53 ± 0.01 ^Ab^	0.57 ± 0 ^Ba^	0.49 ± 0.01 ^Cc^	0.32 ± 0.01 ^Bg^
Glucose (%)									
control	3.96 ± 0 ^ABb^	4.05 ± 0.1 ^Ba^	3.37 ± 0.07 ^Cc^	3.52 ± 0.16 ^Ac^	2.43 ± 0.01 ^Dd^	3.32 ± 0.05 ^Bc^	3.77 ± 0.08 ^ABb^	4.06 ± 0.12 ^Aa^	2.43 ± 0.31 ^Cd^
0.01 mM	3.61 ± 0.37 ^BCc^	4.44 ± 0.06 ^Aa^	4.23 ± 0.07 ^Aab^	3.76 ± 0.05 ^Bc^	4.45 ± 0.24 ^Aa^	3.67 ± 0.16 ^Ac^	3.95 ± 0.33 ^Abc^	3.87 ± 0.11 ^ABc^	2.97 ± 0.04 ^Bd^
0.05 mM	3.99 ± 0.04 ^Aa^	3.54 ± 0.15 ^Cbcd^	3.71 ± 0.11 ^Bbc^	3.46 ± 0.13 ^Bd^	3.41 ± 0.08 ^Cd^	3.57 ± 0.06 ^ABbcd^	3.58 ± 0.1 ^Bbcd^	3.74 ± 0.17 ^Bb^	3.51 ± 0.12 ^Acd^
0.25 mM	3.4 ± 0.05 ^Cb^	3.93 ± 0.22 ^Ba^	3.37 ± 0.11 ^Cb^	2.02 ± 0.08 ^Cd^	3.77 ± 0.04 ^Ba^	3.7 ± 0.31 ^Aa^	3.74 ± 0.04 ^ABa^	3.42 ± 0.01 ^Cb^	2.87 ± 0.03 ^Bc^
Fructose (%)									
control	4.42 ± 0.13 ^Ac^	4.88 ± 0.05 ^Aa^	3.92 ± 0.01 ^Bd^	4.36 ± 0.12 ^Ac^	2.8 ± 0.31 ^Cf^	3.97 ± 0.04 ^Bd^	4.82 ± 0.04 ^ABab^	4.61 ± 0.08 ^Bbc^	3.4 ± 0.22 ^Be^
0.01 mM	3.69 ± 0.19 ^Bd^	4.85 ± 0.13 ^Aab^	5.24 ± 0.25 ^Aa^	4.35 ± 0.19 ^Ac^	4.77 ± 0.26 ^Ab^	4.18 ± 0.27 ^Bc^	5.03 ± 0.33 ^Aab^	4.8 ± 0.08 ^Ab^	3.75 ± 0.21 ^Ad^
0.05 mM	4.39 ± 0.1 ^Aa^	4.01 ± 0.01 ^Bcd^	4.1 ± 0.18 ^Bcd^	4.09 ± 0.12 ^Bcd^	4.04 ± 0.17 ^Bcd^	4.21 ± 0.1 ^Babc^	4.15 ± 0.15 ^Cbcd^	4.37 ± 0.15 ^Cab^	3.95 ± 0.02 ^Ad^
0.25 mM	3.82 ± 0.09 ^Bd^	4.8 ± 0.27 ^Aa^	3.87 ± 0.11 ^Bd^	2.31 ± 0.03 ^Ce^	4.56 ± 0.03 ^Ab^	4.84 ± 0.13 ^Aa^	4.63 ± 0.14 ^Bab^	4.28 ± 0.01 ^Cc^	3.73 ± 0.1 ^Ad^
TSS (%)									
control	13.3 ± 0.78 ^Bc^	14.07 ± 0.55 ^Aab^	14.37 ± 0.06 ^Ca^	14.57 ± 0.47 ^Aa^	14.23 ± 0.23 ^Aa^	13.93 ± 0.06 ^ABabc^	13.5 ± 0.17 ^Abc^	14.3 ± 0.1 ^Aa^	12.37 ± 0.06 ^Ad^
0.01 mM	15.2 ± 0.62 ^Aa^	13.77 ± 0.8 ^Acd^	14.8 ± 0.2 ^Bab^	14.97 ± 0.21 ^Aab^	14.3 ± 0.1 ^Abc^	14.37 ± 0.32 ^Abc^	13.27 ± 0.42 ^Ad^	13.5 ± 0.2 ^Bd^	11.8 ± 0.1 ^Be^
0.05 mM	14.67 ± 0.6 ^Ab^	12.97 ± 0.6 ^Ade^	15.33 ± 0.12 ^Aa^	14.73 ± 0.4 ^Aab^	13.9 ± 0.17 ^Bc^	13.63 ± 0.25 ^Bcd^	13.37 ± 0.4 ^Acd^	13.3 ± 0.1 ^Bcd^	12.47 ± 0.15 ^Ae^
0.25 mM	14.3 ± 0.53 ^ABab^	13.77 ± 0.49 ^Ab^	14.63 ± 0.06 ^Ba^	13.87 ± 0.15 ^Bb^	13.77 ± 0.06 ^Bb^	13.83 ± 0.29 ^Bb^	12.57 ± 0.32 ^Bc^	12.33 ± 0.15 ^Cc^	11.17 ± 0.15 ^Cd^
Malic acid (%)									
contro^l^	2.69 ± 0.15 ^Db^	3.58 ± 0.13 ^Aa^	2.36 ± 0 ^Dc^	2.05 ± 0.03 ^Ad^	1.11 ± 0.01 ^Cg^	1.79 ± 0.02 ^Ae^	1.58 ± 0.01 ^Af^	1.07 ± 0.02 ^Bg^	0.73 ± 0 ^Dh^
0.01 mM	3.46 ± 0.07 ^Ca^	2.81 ± 0.05 ^BCc^	3.07 ± 0.01 ^Ab^	1.99 ± 0.01 ^ABd^	1.54 ± 0.02 ^Ae^	1.47 ± 0.06 ^Bf^	1.6 ± 0.01 ^Ae^	1.06 ± 0.01 ^Bg^	0.97 ± 0.01 ^Ch^
0.05 mM	5.06 ± 0.05 ^Aa^	2.68 ± 0.02 ^Cb^	2.42 ± 0.03 ^Cc^	1.93 ± 0.07 ^Bd^	1.29 ± 0.01 ^Bg^	1.71 ± 0.02 ^Ae^	1.61 ± 0 ^Af^	1.18 ± 0 ^Ah^	1.23 ± 0.01 ^Bh^
0.25 mM	3.79 ± 0.16 ^Ba^	2.86 ± 0.03 ^Bb^	2.49 ± 0 ^Bc^	1.3 ± 0.03 ^Cf^	1.57 ± 0.02 ^Ae^	1.46 ± 0.18 ^Be^	1.31 ± 0.04 ^Bf^	1.01 ± 0.02 ^Cg^	1.64 ± 0.07 ^Ad^
Citric acid (%)									
control	0.09 ± 0 ^Bc^	0.14 ± 0 ^Ba^	0.09 ± 0 ^Bbc^	0.11 ± 0.01 ^Ab^	0.06 ± 0.01 ^Be^	0.08 ± 0.01 ^Bd^	0.1 ± 0 ^Ac^	0.08 ± 0.01 ^Ad^	0.06 ± 0 ^Ae^
0.01 mM	0.09 ± 0.01 ^Bcde^	0.13 ± 0.01 ^Ba^	0.11 ± 0.01 ^Ab^	0.1 ± 0 ^Abc^	0.09 ± 0 ^Ade^	0.08 ± 0 ^Bef^	0.09 ± 0.01 ^ABcd^	0.07 ± 0.01 ^Af^	0.07 ± 0.01 ^Af^
0.05 mM	0.13 ± 0.01 ^Aa^	0.1 ± 0.01 ^Cb^	0.08 ± 0 ^Cde^	0.09 ± 0.01 ^Bbcd^	0.08 ± 0 ^Acde^	0.09 ± 0 ^Abc^	0.09 ± 0.01 ^ABbcd^	0.07 ± 0.01 ^Aef^	0.06 ± 0.01 ^Af^
0.25 mM	0.1 ± 0 ^Bb^	1.09 ± 0.01 ^Aa^	0.09 ± 0 ^Bbc^	0.05 ± 0 ^Cg^	0.09 ± 0 ^Ac^	0.08 ± 0 ^ABde^	0.09 ± 0 ^Bde^	0.08 ± 0 ^Ae^	0.07 ± 0 ^Af^
Acidity (%)									
control	1.03 ± 0.23 ^Abc^	0.74 ± 0.13 ^Ae^	1.27 ± 0.09 ^Aa^	1.16 ± 0.03 ^Aab^	1.01 ± 0.11 ^Abcd^	0.96 ± 0.06 ^Acd^	0.96 ± 0.06 ^Acd^	0.83 ± 0.01 ^Bde^	0.94 ± 0.01 ^Bcd^
0.01 mM	1.11 ± 0.25 ^Aab^	1.06 ± 0.27 ^Aabc^	1.18 ± 0.04 ^Aa^	1.13 ± 0.03 ^Aab^	0.92 ± 0.03 ^Abc^	1.03 ± 0.1 ^Aabc^	0.93 ± 0.05 ^Aabc^	0.82 ± 0.01 ^Bc^	0.99 ± 0.02 ^Aabc^
0.05 mM	1.11 ± 0.13 ^Aabc^	0.98 ± 0.18 ^Acd^	1.2 ± 0.04 ^Aa^	1.17 ± 0.08 ^Aab^	0.94 ± 0.02 ^Acd^	1.04 ± 0.04 ^Aabcd^	1 ± 0.15 ^Abcd^	0.87 ± 0.01 ^Ad^	0.95 ± 0.01 ^Bcd^
0.25 mM	1.12 ± 0.25 ^Aab^	0.86 ± 0.05 ^Acd^	1.23 ± 0.11 ^Aa^	1.03 ± 0.03 ^Bbc^	0.94 ± 0.03 ^Abc^	0.94 ± 0.07 ^Abc^	0.85 ± 0.05 ^Acd^	0.73 ± 0.02 ^Cd^	0.9 ± 0.01 ^Ccd^
pH									
control	3.68 ± 0.25 ^Aa^	3.3 ± 0.02 ^Ad^	3.3 ± 0.02 ^Ad^	3.42 ± 0.03 ^Acd^	3.05 ± 0.03 ^Ae^	3.7 ± 0.01 ^Ca^	3.4 ± 0.01 ^Bcd^	3.6 ± 0.01 ^Aab^	3.52 ± 0.01 ^Abc^
0.01 mM	3.31 ± 0.09 ^Bde^	3.25 ± 0.09 ^Ae^	3.33 ± 0.05 ^Ade^	3.44 ± 0.04 ^Ac^	3.06 ± 0.01 ^Af^	3.76 ± 0.01 ^Aa^	3.39 ± 0.01 ^Bcd^	3.57 ± 0.01 ^Bb^	3.31 ± 0.01 ^Cde^
0.05 mM	3.3 ± 0.02 ^Be^	3.35 ± 0.03 ^Acde^	3.34 ± 0.03 ^Ade^	3.42 ± 0.16 ^Acd^	3.07 ± 0.03 ^Af^	3.72 ± 0.01 ^Ba^	3.44 ± 0.01 ^Acd^	3.58 ± 0.02 ^Bb^	3.45 ± 0.01 ^Bc^
0.25 mM	3.25 ± 0.08 ^Bd^	3.33 ± 0.05 ^Ac^	3.27 ± 0.04 ^Acd^	3.34 ± 0.02 ^Ac^	3.05 ± 0.02 ^Ae^	3.75 ± 0.01 ^Aa^	3.44 ± 0.01 ^Ab^	3.48 ± 0.01 ^Cb^	3.31 ± 0.03 ^Ccd^
TPC (g GAE·kg^−1^)									
control	36.35 ± 2.18 ^Bf^	39.43 ± 0.46 ^BCcd^	42.59 ± 0.47 ^Ba^	38.77 ± 0.79 ^Bde^	40.75 ± 0.83 ^Bbc^	37.87 ± 0.44 ^Bef^	40.86 ± 1.18 ^Ab^	38.88 ± 0.41 ^Ade^	38.59 ± 0.68 ^Ade^
0.01 mM	40.88 ± 1.06 ^Aa^	41.16 ± 0.8 ^Aa^	41.04 ± 0.7 ^Ca^	36 ± 0.28 ^Bc^	38.26 ± 0.53 ^Cb^	31.7 ± 0.15 ^De^	29.33 ± 0.94 ^Cf^	32.86 ± 1.15 ^Bd^	28.9 ± 2.02 ^Cf^
0.05 mM	39.49 ± 1.01 ^Ab^	40.6 ± 0.99 ^Abb^	47.83 ± 0.6 ^Aa^	45.9 ± 3.79 ^Aa^	49.51 ± 1.67 ^Aa^	39.74 ± 1.13 ^Ab^	31.75 ± 1.33 ^Bc^	31.9 ± 1.52 ^BCc^	33.82 ± 1.44 ^Bc^
0.25 mM	38.86 ± 0.96 ^Ab^	38.41 ± 1.32 ^Cb^	38.61 ± 0.84 ^Db^	45.14 ± 1.18 ^Aa^	35.56 ± 0.46 ^Dc^	35.55 ± 0.52 ^Cc^	23.84 ± 0.28 ^De^	30.38 ± 0.19 ^Cd^	22.93 ± 1.34 ^De^
TAC (g C3 GE·kg^−1^)									
control	10.04 ± 0.2 ^Dd^	13.97 ± 0.77 ^Ab^	18.1 ± 0.09 ^Ba^	14.5 ± 0.22 ^Ab^	9.91 ± 0.02 ^Dd^	12.76 ± 0.06 ^Bc^	10.12 ± 0.25 ^Bd^	8.72 ± 0.23 ^Ae^	9.06 ± 0.15 ^Ae^
0.01 mM	14.38 ± 0.37 ^Aa^	13.02 ± 0.68 ^Abb^	15.43 ± 0.31 ^Ca^	11.94 ± 0.69 ^Cb^	12.08 ± 0 ^Bb^	7.17 ± 0.26 ^Dd^	9.5 ± 0.36 ^Cc^	6.94 ± 1.28 ^Bd^	2.81 ± 0.08 ^Ce^
0.05 mM	12.12 ± 0.07 ^Cd^	12.66 ± 0.15 ^Bc^	18.79 ± 0.4 ^Aa^	13.39 ± 0.17 ^Bb^	13.14 ± 0.06 ^Ab^	13.34 ± 0.04 ^Ab^	10.6 ± 0.23 ^Ae^	6.79 ± 0.18 ^Bf^	3.91 ± 0.11 ^Bg^
0.25 mM	13.44 ± 0.37 ^Ba^	13.52 ± 0.23 ^Aba^	13.48 ± 0.45 ^Da^	10.23 ± 0.39 ^Dc^	11.3 ± 1.27 ^Cb^	11.74 ± 0.22 ^Cb^	4.48 ± 0.1 ^De^	6.86 ± 0.37 ^Bd^	0.97 ± 0.05 ^Df^
TFC (g CE·kg^−1^)									
control	86.73 ± 2.91 ^Bb^	88.64 ± 0.9 ^Bb^	99.61 ± 1.14 ^Ba^	99.78 ± 3.48 ^Ca^	64.24 ± 5.4 ^Cd^	79.81 ± 2.34 ^Bc^	78 ± 3.38 ^Ac^	60.22 ± 1.76 ^Ad^	52.82 ± 3.48 ^Ae^
0.01 mM	97.5 ± 8.87 ^Ac^	99.11 ± 2.68 ^Ac^	119.9 ± 5.35 ^Aa^	109.4 ± 7.45 ^Bb^	84.42 ± 3.76 ^Ad^	63.67 ± 1.2 ^De^	56.48 ± 1.29 ^Cef^	49.16 ± 1.96 ^Bf^	28.27 ± 0.2 ^Bg^
0.05 mM	94.64 ± 6.01 ^Abc^	83.19 ± 2.27 ^Cd^	100.92 ± 5.28 ^Bc^	139.21 ± 2.34 ^Aa^	73.56 ± 1.53 ^Be^	123.45 ± 1.82 ^Ab^	63.87 ± 1.34 ^Bf^	56.28 ± 3.64 ^Ag^	30.91 ± 3.04 ^Bh^
0.25 mM	93.52 ± 8.07 ^Aba^	80.45 ± 1.05 ^Cb^	95.06 ± 4.38 ^Ba^	95.7 ± 4.94 ^Ca^	71.51 ± 5.17 ^Bc^	72.45 ± 2.85 ^Cc^	44.97 ± 3.17 ^Dd^	48.71 ± 7.79 ^Bd^	32.2 ± 4.36 ^Be^
DPPH (g TE·kg^−1^)									
control	138.09 ± 4.11 ^Bd^	117.55 ± 6.86 ^Be^	162.06 ± 1.36 ^Cc^	93.87 ± 8.5 ^Cf^	113.63 ± 5.47 ^De^	72.04 ± 5.61 ^Dg^	152.13 ± 10.82 ^Ccd^	203.34 ± 5.9 ^Bb^	292.2 ± 1.14 ^Ca^
0.01 mM	166.21 ± 9.46 ^Ade^	126.47 ± 13.52 ^Bf^	188.69 ± 10.38 ^Bc^	153.95 ± 5.49 ^Bde^	140.26 ± 4.33 ^Cef^	230.58 ± 9.69 ^Ab^	240.31 ± 4.25 ^Ab^	199.3 ± 15.47 ^Bc^	335.46 ± 8.91 ^Aa^
0.05 mM	103.45 ± 1.44 ^Ch^	129.22 ± 9.72 ^Bg^	179.1 ± 8.96 ^Bf^	246.02 ± 8.1 ^Ac^	196.17 ± 1.08 ^Ae^	217.14 ± 1.48 ^Bd^	244.41 ± 3.02 ^Ac^	286.03 ± 20.45 ^Ab^	311.18 ± 1.03 ^Ba^
0.25 mM	133.62 ± 2.67 ^Bf^	189.1 ± 5.64 ^Acd^	201.3 ± 10.02 ^Ac^	170.97 ± 17.65 ^Be^	180.9 ± 5.84 ^Bde^	197.01 ± 6.56 ^Cc^	232.54 ± 4.05 ^Bb^	200.72 ± 13.29 ^Bc^	315.01 ± 3.35 ^Ba^
ABTS (g TE·kg^−1^)									
control	10.15 ± 0.05 ^Cf^	8.7 ± 0.02 ^Dg^	10.74 ± 0.04 ^Be^	10.19 ± 0.03 ^Df^	12.03 ± 0.02 ^Ab^	11.08 ± 0.01 ^Bd^	10.78 ± 0.03 ^Ce^	11.43 ± 0.04 ^Dc^	14 ± 0.06 ^Ba^
0.01 mM	10.09 ± 0.06 ^Ce^	9.29 ± 0.17 ^Cf^	9.23 ± 0.04 ^Df^	11 ± 0.07 ^Cd^	10.94 ± 0.16 ^Cd^	11.93 ± 0.07 ^Ac^	11.83 ± 0.03 ^Bc^	12.48 ± 0.03 ^Cb^	14.06 ± 0.04 ^Bb^
0.05 mM	10.9 ± 0.04 ^Af^	11.37 ± 0.08 ^Ae^	9.63 ± 0.01 ^Cg^	12.5 ± 0.04 ^Bb^	11.92 ± 0.09 ^Ad^	10.94 ± 0.05 ^Cf^	12.31 ± 0.01 ^Ac^	12.97 ± 0.08 ^Ba^	12.44 ± 0.08 ^Cb^
0.25 mM	10.77 ± 0.03 ^Bg^	9.97 ± 0.15 ^Bh^	10.98 ± 0.13 ^Af^	19.55 ± 0.03 ^Aa^	11.67 ± 0.34 ^Be^	11.16 ± 0.11 ^Bf^	12.33 ± 0.01 ^Ad^	14.26 ± 0.08 ^Ac^	15.78 ± 0.05 ^Ab^
FRAP (g FeSO_4_.7 H_2_O·kg^−1^)									
control	241.69 ± 2.56 ^Ca^	136.33 ± 3.06 ^Dg^	163.66 ± 1.39 ^Cc^	157.47 ± 4.47 ^Cde^	161.49 ± 6.45 ^Ccd^	175.81 ± 2.73 ^Bb^	151.13 ± 4.24 ^Def^	147.81 ± 9.65 ^Cf^	165.16 ± 2.85 ^Bc^
0.01 mM	244.74 ± 13.47 ^Cc^	295.1 ± 15.29 ^Aab^	284.3 ± 7.19 ^Aab^	284.03 ± 8.19 ^Aab^	302.28 ± 6.61 ^Ba^	192.83 ± 16.94 ^Bd^	255.09 ± 6.05 ^Bc^	251.9 ± 1.25 ^Ac^	187.92 ± 2.49 ^Ad^
0.05 mM	296.93 ± 1.45 ^Aab^	250.17 ± 21.3 ^Bde^	269 ± 12.37 ^Bcd^	294.91 ± 17.92 ^Aab^	315.36 ± 8.95 ^Aa^	276.25 ± 21.42 ^Abc^	287.77 ± 7.99 ^Abc^	227.06 ± 12.29 ^Be^	89.92 ± 8.01 ^Df^
0.25 mM	276.74 ± 8.62 ^Bc^	213.62 ± 13.07 ^Cd^	264.32 ± 6.68 ^Bc^	209.35 ± 2.33 ^Bd^	311.95 ± 8.15 ^Aa^	294.02 ± 3.08 ^Ab^	195.75 ± 7.23 ^Ce^	217.99 ± 4.5 ^Bd^	121.32 ± 12.53 ^Cf^
MDA (mmol·kg^−1^)									
control	125.77 ± 9.99 ^Cd^	256.4 ± 20.64 ^Ab^	339.11 ± 4.07 ^Aa^	128.67 ± 14.54 ^Ad^	71.75 ± 11.56 ^Be^	166.12 ± 5.04 ^Ac^	115.37 ± 16.83 ^Bd^	82.7 ± 4.83 ^Ae^	42.65 ± 1.27 ^Af^
0.01 mM	280 ± 39.84 ^Aa^	194.64 ± 3.95 ^Bb^	262.92 ± 2.75 ^Ba^	114.74 ± 3.09 ^ABc^	120.67 ± 39.75 ^ABc^	78.96 ± 12.19 ^Bd^	120.21 ± 4.79 ^Bc^	50.46 ± 2.85 ^Bd^	6.06 ± 0.58 ^Ce^
0.05 mM	202.65 ± 6.62 ^Bb^	175.02 ± 6.95 ^Bc^	236.5 ± 17.22 ^Ca^	109.12 ± 4.78 ^Be^	132.83 ± 24.97 ^Ade^	170.14 ± 30.57 ^Ac^	138.88 ± 2.01 ^Ad^	35.99 ± 0.84 ^Cf^	22.53 ± 1.23 ^Bf^
0.25 mM	136.41 ± 9.77 ^Cab^	137.68 ± 8.7 ^Cab^	157.57 ± 17.18 ^Da^	68.18 ± 1.47 ^Cc^	117.66 ± 28.9 ^ABb^	111.94 ± 36.11 ^Bb^	57.28 ± 1.83 ^Ccd^	35.61 ± 1.19 ^Cd^	1.18 ± 0.09 ^De^

Note: GAE: gallic acid equivalent; CE: catechin equivalent; C3GE: cyanidin-3-glucoside equivalent. Different capital letters indicate significant differences among treatments at the same storage time (*p* < 0.05), while different lowercase letters indicate significant differences among storage times within the same treatment (*p* < 0.05).

**Table A2:** Quantification of polyphenol compounds of blue honeysuckle (mg/100 g FW).

Bioactive Components	0 Day	63 Day			
	Control	Control	0.01 mM	0.05 mM	0.25 mM
Anthocyanins					
Cyanidin-3,5-diglucoside	0.04 ± 0	0.03 ± 0.01	0.04 ± 0	0.04 ± 0	0.03 ± 0
Peonidin-3,5-diglucoside	51.7 ± 0.16	51.78 ± 0.95	51.88 ± 0.64	52 ± 0.32	51.81 ± 0.5
Cyanidin-3-glucoside	400.74 ± 0.45	345.02 ± 1.3	67.4 ± 0.29	79.83 ± 0.79	54.13 ± 1.24
Cyanidin-3-rutinoside	51.52 ± 0.11	51.35 ± 0.38	51.8 ± 0.29	ND	51.81 ± 1.15
Pelargonidin-3-glucoside	51.43 ± 0.13	51.68 ± 0.64	51.21 ± 0.29	51.37 ± 1.17	50.7 ± 0.51
Peonidin-3-glucoside	58.68 ± 0.26	52.16 ± 1.1	52 ± 0.7	51.51 ± 0.63	52.11 ± 1.68
Peonidin-3-rutinoside	51.57 ± 0.1	50.75 ± 0.84	51.6 ± 0.37	52.4 ± 2.44	51.83 ± 0.69
Cyanidin-3-xyloside	51.54 ± 0.31	51.82 ± 0.46	ND	ND	ND
Peonidin-3-glucoside pyruvic derivative	51.5 ± 0.22	ND	ND	ND	ND
Delphinidin-3-glucoside	51.4 ± 0.54	51.53 ± 0.37	51.48 ± 0.18	ND	51.61 ± 1.85
Delphinidin-3-sambubioside	51.55 ± 0.13	51.51 ± 0.2	51.15 ± 0.1	ND	51.26 ± 1.78
Delphinidin-3-rutinoside	57.13 ± 0.89	51.87 ± 0.06	50.1 ± 0.61	51.48 ± 0.13	51.4 ± 0.28
Phenolic acids					
Gallic acid	6.66 ± 1.58	3.93 ± 0.07	ND	ND	ND
Ellagic acid acetyl-xyloside	5.14 ± 0.35	4.01 ± 0.25	7.11 ± 0.26	4.12 ± 0.11	3.37 ± 1.32
Quinic acid	13.57 ± 0.14	8.49 ± 0.12	9.04 ± 1.07	8.15 ± 0.27	4.59 ± 0.83
p-Coumaric acid ethyl ester	37.93 ± 1.1	24.75 ± 0.06	22.93 ± 2.15	19.25 ± 1.03	8.43 ± 0.04
p-Coumaroylquinic acid	4.79 ± 0.12	ND	2.32 ± 0.94	3.14 ± 1.26	ND
Caffeoyl glucose	1.45 ± 0.28	ND	ND	0.53 ± 0.01	ND
5-O-Caffeoylquinic acid	1.61 ± 0.35	0.98 ± 0	4 ± 0.25	4.36 ± 0.93	ND
Neochlorogenic acid	0.46 ± 0.04	26.85 ± 0.19	ND	1.31 ± 0.08	ND
Chlorogenic acid	65.84 ± 0.26	108.01 ± 0.41	ND	ND	ND
3-O-Caffeoylquinic acid	ND	ND	0.7 ± 0.26	1.11 ± 0.5	ND
3-Feruloylquinic acid	25.62 ± 0.42	26.69 ± 0.36	6 ± 0.89	3.62 ± 0.76	4 ± 0.61
Trihydroxycinnamoylquinic acid (isomer)	5.6 ± 0.27	3.6 ± 0	0.97 ± 0	0.87 ± 0.04	ND
Dicaffeoylquinic acid	2.98 ± 0.22	3.51 ± 0.03	2.83 ± 0.67	6.59 ± 0.63	ND
Dihydrocaffeic acid	1.17 ± 0.23	1.92 ± 0.05	0.99 ± 0	0.75 ± 0.49	ND
Flavonoids					
Phloretin 2′-O-xylosyl-glucoside	47.51 ± 0.16	40.18 ± 0.3	ND	28.01 ± 0.16	ND
Flavanols					
(+)-Catechin	0.7 ± 0.01	4.07 ± 0.26	ND	4.33 ± 0.47	ND
(-)-Gallocatechin	6.91 ± 0.32	2.96 ± 0.54	ND	ND	ND
Flavanones					
Eriodictyol 7-O-glucoside	29.39 ± 1.07	225.82 ± 0.44	10.32 ± 1.4	16.7 ± 0.61	29.47 ± 0.14
Flavones					
Luteolin 7-O-glucoside	ND	ND	ND	24.2 ± 2.11	ND
Luteolin 7-O-rutinoside	0.01 ± 0	11.42 ± 0.27	ND	8.09 ± 1.92	ND
Luteolin-O-hexose-O-deoxyhexoside	21.09 ± 0.45	22.62 ± 0.55	ND	ND	ND
Flavonols					
Quercetin-3-O-galactoside	40.79 ± 0.51	6.18 ± 0.46	1.4 ± 0	33.09 ± 0.64	ND
Myricetin 3-O-rhamnoside	36.5 ± 0.06	ND	ND	ND	ND
Quercetin 3-*O*-glucoside	40.16 ± 1.49	ND	ND	ND	ND
Quercetin- acetyl -hexoside	38.9 ± 0.56	96.84 ± 1.63	9.1 ± 0.11	ND	ND
Kaempferol 3-O-galactoside 7-O-rhamnoside	320.48 ± 1.9	55.45 ± 0.36	26.49 ± 0.49	17.27 ± 0.52	ND
Kaempferol 3-O-rutinoside	372.61 ± 0.80	310.64 ± 0.26	ND	1.77 ± 0.89	ND
Quercetin *O*-vicianoside	62.91 ± 1.09	26.97 ± 0.2	1.14 ± 0.26	30.3 ± 1.96	ND
Quercetin-3-hexoside-pentoside	157.12 ± 0.39	88.76 ± 0.39	ND	ND	ND
Quercetin-3-O-rhamnosiy-galactoside	322.97 ± 0.63	13.15 ± 0.09	9.18 ± 0.09	41.44 ± 0.53	17.83 ± 0.65
Quercetin 3-*O*-rutinoside	5.38 ± 0.42	59.16 ± 0.17	30.58 ± 0.26	35.06 ± 2.23	24.33 ± 0.64
Kaempferol 3,7-O-diglucoside	119.41 ± 0.79	172.68 ± 0.42	ND	95.08 ± 0.09	ND
Isorhamnetin-3-O-rutinoside	10.75 ± 0.53	11.28 ± 0.94	ND	ND	ND
Quercetin 3-O-sophoroside	0.01 ± 0	2.91 ± 0.04	ND	ND	ND
Quercetin 7,4′-O-diglucoside	9.86 ± 0.51	ND	ND	ND	ND
Quercetin 3,4′-*O*-dihexoside	66.52 ± 0.28	ND	ND	ND	ND
Myricetin-3-O-rutinoside	62.12 ± 0.49	ND	ND	ND	ND
Isoflavonoids					
Genistin	ND	100.62 ± 0.65	ND	ND	ND
Other polyphenols					
p-Anisaldehyde	3.23 ± 0.48	3.59 ± 0.77	0.6 ± 0.04	1.37 ± 0.07	ND
Hydroxytyrosol	1.37 ± 0.01	4.06 ± 0.61	2.01 ± 0.12	6.17 ± 1.48	3.5 ± 0.85
5-Nonadecylresorcinol	ND	ND	2.62 ± 0.05	1.62 ± 0.33	4.79 ± 0.07
5-Heneicosylresorcinol	7.87 ± 0.62	2.73 ± 0.51	3.27 ± 0.66	ND	2.27 ± 0.55

**Table A3:** Correlation analysis among postharvest quality of blue honeysuckle during storage.

	l	a	b	Chroma (C)	HUE	ΔE	Weight Loss	Firmness	Sucrose	Maltose	Glucose	Fructose	Total Sugar	TSS	Malic Acid	Citric Acid	Total Acid	Acidity	pH	TPC	TAC	TFC	DPPH	ABTS	FRAP	MDA
l		*	*	*	*	*	*			*					*		*									
a	−0.43		*	*	*	*	*	*						*						*	*	*	*	*		*
b	−0.66	0.8		*	*	*	*					*		*						*	*	*	*	*		*
Chroma(C)	0.69	−0.72	−0.99		*	*								*						*	*	*	*	*		*
Hue	0.26	−0.75	−0.54	0.51		*		*															*			
ΔE	0.97	−0.38	−0.65	0.68	0.22		*			*					*	*	*									
Weight loss	0.2	0.26	0.21	−0.18	−0.17	0.21				*	*			*	*	*	*	*	*	*	*	*	*	*		*
Firmness	0.12	−0.23	−0.15	0.12	0.33	0.13	−0.071				*	*	*					*								
Sucrose	−0.091	0.087	0.056	−0.06	−0.024	−0.1	−0.099	0.011			*	*	*		*	*	*		*					*	*	
Maltose	0.25	−0.12	−0.081	0.071	0.017	0.25	0.42	−0.18	0.05				*		*	*	*		*	*	*	*				*
Glucose	0.061	−0.14	−0.15	0.13	0.032	0.013	−0.21	0.22	0.34	0.05		*	*	*	*		*				*	*	*	*	*	*
Fructose	0.12	−0.14	−0.19	0.18	0.09	0.076	−0.038	0.29	0.37	0.092	0.85		*		*	*	*	*		*				*	*	*
Total sugar	0.094	−0.13	−0.15	0.13	0.054	0.047	−0.081	0.21	0.56	0.2	0.92	0.94			*	*	*							*	*	*
TSS	−0.065	−0.27	−0.28	0.24	−0.053	−0.058	−0.65	−0.074	−0.017	−0.014	0.29	0.1	0.17		*		*	*		*	*	*	*	*	*	*
Malic acid	−0.31	−0.12	−0.028	−0.0069	0.12	−0.33	−0.75	0.16	0.26	−0.4	0.37	0.19	0.26	0.46		*	*	*	*	*	*	*	*	*	*	*
Citric acid	−0.15	0.031	0.1	−0.12	0.086	−0.19	−0.23	0.082	0.23	−0.25	0.19	0.22	0.21	0.042	0.24		*							*		
Total acid	−0.32	−0.11	−0.0094	−0.027	0.12	−0.34	−0.74	0.17	0.28	−0.42	0.38	0.22	0.28	0.44	0.99	0.4		*	*	*	*	*	*	*	*	*
Acidity	−0.15	0.015	−0.0029	0.0062	−0.15	−0.17	−0.41	−0.21	0.035	−0.15	−0.05	−0.19	−0.12	0.5	0.34	−0.13	0.3			*	*	*		*	*	*
pH	−0.046	0.074	0.041	−0.051	−0.12	−0.054	0.22	−0.05	−0.22	0.23	0.043	0.15	0.066	−0.13	−0.22	−0.077	−0.22	−0.13		*	*					*
TPC	−0.031	−0.31	−0.29	0.27	0.086	−0.028	−0.61	−0.1	−0.056	−0.29	−0.033	−0.19	−0.15	0.63	0.31	0.051	0.3	0.38	−0.27		*	*	*	*	*	*
TAC	−0.076	−0.35	−0.35	0.31	0.018	−0.079	−0.77	−0.0074	0.11	−0.29	0.25	0.11	0.15	0.75	0.54	0.17	0.54	0.48	−0.21	0.78		*	*	*	*	*
TFC	−0.037	−0.37	−0.37	0.34	0.14	−0.025	−0.68	−0.037	0.087	−0.21	0.22	0.061	0.12	0.72	0.54	0.07	0.53	0.55	−0.085	0.69	0.82		*	*	*	*
DPPH	0.045	0.36	0.3	−0.24	−0.27	0.064	0.81	−0.15	−0.18	0.14	−0.24	−0.094	−0.17	−0.58	−0.53	−0.064	−0.51	−0.18	0.18	−0.49	−0.64	−0.54		*	*	*
ABTS	−0.018	0.21	0.32	−0.31	−0.011	0.022	0.6	−0.1	−0.27	0.18	−0.71	−0.62	−0.64	−0.51	−0.55	−0.23	−0.56	−0.21	0.042	−0.28	−0.6	−0.43	0.49		*	*
FRAP	−0.16	−0.081	0.041	−0.063	−0.074	−0.17	−0.18	−0.16	0.21	0.15	0.36	0.26	0.34	0.39	0.28	−0.0044	0.26	0.24	−0.19	0.28	0.42	0.48	−0.2	−0.23		*
MDA	−0.12	−0.26	−0.25	0.23	2.4 × 10^−4^	−0.13	−0.75	0.094	0.098	−0.37	0.36	0.21	0.23	0.62	0.69	0.092	0.67	0.42	−0.2	0.55	0.85	0.68	−0.56	−0.66	0.28	

Note: “*” represents *p* ≤ 0.05, TOC.
